# Early ontogeny and sequence heterochronies in Leiuperinae frogs (Anura: Leptodactylidae)

**DOI:** 10.1371/journal.pone.0218733

**Published:** 2019-06-27

**Authors:** Jimena Grosso, Diego Baldo, Darío Cardozo, Francisco Kolenc, Claudio Borteiro, Marianna I. R. de Oliveira, Marcelo F. Bonino, Diego A. Barrasso, Florencia Vera Candioti

**Affiliations:** 1 Unidad Ejecutora Lillo (CONICET-FML), Tucumán, Argentina; 2 Laboratorio de Genética Evolutiva, (IBS-CONICET), Misiones, Argentina; 3 Sección Herpetología, Museo Nacional de Historia Natural, Montevideo, Uruguay; 4 Programa de Pós-Graduação em Biodiversidade e Evolução (PPGBioEvo-UFBA), Ondina, Salvador, Bahia, Brazil; 5 Laboratorio de Ecología, Biología Evolutiva y Comportamiento de Herpetozoos (LEBECH), INIBIOMA (CONICET-UNCo), Rio Negro, Argentina; 6 Instituto de Diversidad y Evolución Austral (IDEAus-CONICET), Chubut, Argentina; 7 Facultad de Ciencias Naturales y Ciencias de la Salud, Universidad Nacional de la Patagonia “San Juan Bosco” (UNPSJB), Chubut, Argentina; Laboratoire de Biologie du Développement de Villefranche-sur-Mer, FRANCE

## Abstract

The study of early development in Neotropical Leiuperinae frogs (Anura, Leptodactylidae) has been addressed by several works in recent times. However, a comparative developmental approach under a phylogenetic context was not available. Herein we analyzed the morphological and ontogenetic diversity of embryos belonging to 22 species of the three largest genera in Leiuperinae. We find that in most cases, variations fit with the phylogeny at the inter- and intrageneric levels. Embryo kyphosis and whitish color are synapomorphies for the clade grouping *Physalaemus* and *Engystomops*. The presence of a third lower tooth row on the oral disc is plesiomorphic for Leiuperinae, only changing in derived clades. The configurations and developmental trajectories of the lower lip are exceptionally diverse. The developmental sequences optimized on the phylogenetic tree recover an early differentiated first lower tooth row a synapomorphy of *Pseudopaludicola* and *Physalaemus*, and an early differentiated second row as synapomorphy of *Pleurodema*. On the other hand, few features are highly conserved in the subfamily, such as the adhesive glands universally present in a type-C configuration. Our results also suggest that the morphology and ontogeny of embryos is in some cases associated to the environment where they develop. A large body size, poorly developed transient respiratory structures, large yolk provision and delayed development of the digestive tract occur convergently in embryos inhabiting cold, oxygenated environments. Embryos that develop in warmer water bodies in xeric environments show more complex and persistent transient respiratory structures and an early onset of hind limbs development. Our survey highlights that morphology and early development of anurans can be a valuable source of information for phylogenetic studies, and provide fundamental bases to explore and discuss how evolutionary changes can be shaped by environmental conditions.

## Introduction

The Neotropical group Leiuperinae is one of the three major clades of the anuran family Leptodactylidae (sensu [1]), and as currently defined by the latest phylogenetic hypotheses it is composed of the genera *Edalorhina*, *Engystomops*, *Physalaemus*, *Pleurodema*, and *Pseudopaludicola* [1–3]. The subfamily includes 99 small-sized frogs commonly known as Foam, Dwarf, and Four-eyed frogs [4]. These species vary in several aspects of their reproductive biology, with at least four developmental modes that differ in oviposition site, clutch structure, and types of environments for tadpole development.

The oviposture in foam nests on the surface of ponds is the widespread oviposition type in the group. All species of *Edalorhina*, *Engystomops*, and *Physalaemus* lay eggs in single or communal foam nests on lentic water bodies (e.g., [5–6]), and even on the water accumulated on the axils of bromeliads in the case of *Ph*. *spiniger* [7]. In *Pleurodema* the foam nest is the plesiomorphic state and occurs in most species [8–10], with a curious variation in *Pl*. *tucumanum* where the foam soon collapses [11]. Additionally, other reproductive modes were described for leiuperines. The foam nest is lost in a derived *Pleurodema* clade joining the *Pl*. *bibroni* and *Pl*. *thaul* clades. Species of *Pl*. *bibroni*, *Pl*. *cordobae*, and *Pl*. *kriegi* lay a few eggs embedded in a subspherical gelatinous mass [12–13], whereas *Pl*. *thaul*, *Pl*. *bufoninum*, and presumably *Pl*. *somuncurensis* ovipose in gelatinous strings [14–16]. Species of *Pseudopaludicola* lay individual eggs or small groups of loosely attached eggs at the bottom of shallow ponds (e.g., [17–19]).

Geographic distribution of leiuperine species has a wide latitudinal range, from Southern Mexico to Argentinean and Chilean Patagonia. Thus, oviposition occurs in a variety of environmental conditions. Species that inhabits xeric environments, like the Brazilian Caatinga and the South American Chaco, breed in highly ephemeral ponds and present an explosive reproductive activity triggered by heavy rainfall in the warm season (e.g., [9–10, 20]). On the other extreme, the most austral species of *Physalaemus* and the clade joining *Pleurodema bibroni* and *Pl*. *thaul* breed on ponds and flooded grassland at high latitudes or during the cold season in asynchrony with the reproductive events of sympatric species (e.g., [12–13, 15–16, 21–22]).

Several studies summarized characteristics of the early development in leiuperine species. In *Engystomops*, a table of normal development was proposed [23], and details of gastrulation and ontogeny of transient embryonic structures are available [24–29]. Developmental tables are also described for three species of *Pleurodema* [30–32]. In *Physalaemus*, the ontogeny from blastula to metamorphosis has been synthesized for *Ph*. aff. *albonotatus* (as *Ph*. *albonotatus*, [33]), as well as some aspects of transient structures in *Ph*. *biligonigerus* [34]. In *Pleurodema*, a brief description of the first larval stages and development time was given for *Pl*. *bufoninum* [16]. Finally, a comparative analysis of the oral disc ontogeny in 12 species of *Physalaemus*, *Pleurodema*, and *Pseudopaludicola* reveals heterochronic variations related to interspecific differences in oral morphology [35]. While all these studies are very informative, a comparative study framed in an explicit phylogenetic context has not been attempted yet.

Here we study the early ontogeny in three genera of leiuperine frogs, including species with different types of clutches, and varied sites of oviposition and tadpole development. Our main goals are to survey the morphological diversity in embryonic characters and explore heterochronic variations in their developmental sequences, in order to interpret evolutionary transformations along the history of the subfamily.

## Material and methods

We analyzed developmental series of Leiuperinae frogs in the period between the differentiation of the tail bud and the full development of the oral disc and/or hind limb emergence. Gosner stages [36] are not particularly useful in this period, because stages are often hard to identify in preserved material, and most importantly, because there is a high uncoupling of characters defining stages (discussed in [37]. Consequently, we refer to Gosner stages (now on GS) only in a few cases when pertinent. We sampled 22 species belonging to the genera *Physalaemus* (12 species of the *Ph*. *cuvieri* clade), *Pleurodema* (8 species of 4 clades), and *Pseudopaludicola* (2 species from two clades) (S1 Appendix). Ontogenetic series were obtained from clutches collected in the field under permission of national and regional authorities: Argentina, Dirección de Flora y Fauna (81/2015 and 01/2017 –DS y FS), Secretaría de Medioambiente (N° 41-AP-2014), Dirección Provincial de Áreas Naturales Protegidas (14/16), Secretaria de Ambiente from Ministerio de Ambiente y Producción Sustentable (EXPTE 0090227-13596/2014-0), Dirección Flora, Fauna Silvestre y Suelos (EXPTE 1865-330-P-2,015), Ministerio de Ecología y Recursos Naturales Renovables (MEyRNR, 007/2009, 048/2013, 072/2014, 061/2015, 073/2016, and 035/2017), Programa de Recursos Naturales y Medio Ambiente (PRNyMA, 01/2016); Brazil, SISBIO (N.° 60078–3 Código de autenticação: 0600780320181101); Uruguay, División Fauna, Ministerio de Ganadería, Agricultura y Pesca (Res. N° 199/13 and 137/16). The specimen manipulation was carried out following the recommendations in the Guidelines for Ethical Conduct in the Care and Use of Nonhuman Animals in Research (CARE) and the CEUA-MNHN protocol (Res. 1/2019). Embryos were periodically (every 6–8 hs) euthanized by immersion in water with lidocaine (AVMA Guidelines for the Euthanasia of Animal, 2013 Edition), and preserved in 10% buffered formalin. Between fixations, embryos were kept under seminatural conditions at or near the place where clutches were collected; in the few cases where embryos were taken to the laboratory, no controlled, standardized conditions were maintained. Species identity was confirmed by one or more criteria, which include identification of amplectant pairs, rearing some specimens through metamorphosis, unique geographic distributions, and clutch structure. We examined series from 85 clutches, approximately totaling 4500 embryos. Specimens were observed, measured, and photographed with a Leica M205 stereomicroscope. Additionally, 5–12 embryos of all taxa were dehydrated using serial dilutions of ethanol and coated with gold to perform scanning with a Zeiss Supra 55VP electron microscope. Micrographs were taken mainly from a ventral view because of the arrangement of most morphological structures. Transient embryonic structures (i.e., epidermal ciliated cells, external gills, and adhesive and hatching glands) were characterized following previous studies [25–29]. The larval oral disc and its developmental stages were described following Altig [38] and Thibaudeau and Altig [39] terminology; the definitive configuration was determined by comparison with tadpole descriptions already published for each species (e.g., [35] and citations therein; [40–41]). Embryo measurements were obtained from photographs taken under the stereomicroscope and analyzed with Leica Application Suite software (V4.4.0), and include the embryo body length and area, yolk area at tailbud stage, extent of dorsal curvature at tailbud stage (measured in lateral view, as the angle subtended by the embryos body from a dorsal midpoint), height and diameter of fully developed adhesive glands, length of primary filaments of first and second gill pairs, average length of secondary filaments of the two first gill pairs, and average length of all filaments of the third gill pair if present. All measurements and structural and nomenclatural details of transient structures are illustrated in S1 Fig.

To study changes in developmental timing under the approach of sequence heterochrony [42], we synthesized ontogenetic sequences for each species by defining a maximum of 34 events related to ontogeny of the tail, gills, oral disc and digestive tract, hind limbs, and adhesive glands; we also included two events that were intraspecifically variable, concerning the hatching time and the emergence of labial teeth (Table 1). This information was used to construct complete developmental sequences for each species, considering events that were not universally present across samples.

**Table 1:** Events in Leiuperinae early development. These events, here listed by morphological structure, were used to construct summarized developmental sequences per species, thus some may be missing in some taxa.

Developmental events	
**Tail**	
(1)	tail bud
(2)	caudal fin buds
(3)	tail length / body length = 1
(4)	tail length / body length = 2
**Gills**	
(5)	first gill pair bud
(6)	first gill pair branched
(7)	second gill pair bud
(8)	second gill pair branched
(9)	third gill pair bud
(10)	operculum at gill base
(11)	gills at full development
(12)	operculum medially fused
(13)	right gill covered by operculum
(14)	left gill covered by operculum
(15)	spiracle developed
**Oral disc and digestive tract**	
(16)	labial tooth ridge A1
(17)	labial tooth ridge A2
(18)	labial tooth ridge P1
(19)	labial tooth ridge P2
(20)	labial tooth ridge P3
(21)	marginal papillae at commissures
(22)	marginal papillae medial in mental region
(23)	marginal papillae lateral in mental region
(24)	marginal papillae with larval configuration
(25)	oral disc fully formed (i.e., all mouthparts present at least as anlagen)
(26)	first coil in the digestive tract
(27)	onset of active feeding
**Hind limbs**	
(28)	hind limb buds (as small protuberances at the tail base)
(29)	hind limbs at GS26
**Adhesive glands**	
(30)	adhesive glands first visible
(31)	adhesive glands separated
(32)	adhesive glands absent
**Intraspecifically variable**	
(33)	hatching
(34)	emergence of the first labial teeth

Comparisons among species were performed through sequence heterochrony plots [42]. In this case, a subset of 24 comparable events was used, excluding those related to the presence of a third gill pair (9), a third lower labial ridge (20), and adhesive gland division (31), events with low variation in the order of sequence (2, 4), and events with high intraspecific variations (33, 34). We also excluded the onset of active feeding (27) because we are not certain about the exact timing in some cases. To allow a comparison between species with very different oral trajectories, events concerning marginal papillae (21–24) were condensed in first marginal papillae differentiation and marginal papillae with the larval configuration. The sequence of events recorded for each species was converted into an ordering rank, 1–24 from the earliest to the latest; synchronous events were given the mean rank for all the events that occurred at that time. The sequences for each species were further checked by examining embryos at random from the same and different clutches. In some cases we could only collect a single clutch, which prohibited fully assessing intraspecific variations more than at an intraclutch level. Developmental events of all species were plotted against their ranks using a developmental sequence of *Odontophrynus americanus* as a reference trajectory. In this approach, heterochronic shifts are the changes in the relative position or events regarding those in the reference trajectory. Although we name these changes in timing terminology (e.g., accelerated/delayed, fast/slow, short/long development), absolute time is not explicitly considered in this context.

Finally, we performed a maximum parsimony-based optimization of morphological characters and ontogenetic sequences, on a meta-tree that combined the phylogenetic hypotheses by Pyron [43], de Sá et al. [44] for *Leptodactylus*, Faivovich et al. [45] for *Pleurodema*, Lourenço et al. [46] for *Physalaemus*, and Veiga-Menoncello et al. [47] for *Pseudopaludicola*. The software TNT version 1.5 [48] was used to reconstruct ancestral states of morphological features coded for 3 continuous and 6 discrete characters: (1) body length at tailbud stage; (2) angle of dorsal curvature at tailbud stage; (3) yolk area relative to body area; (4) embryo pigmentation: present, absent; (5) adhesive gland: type C, type D, absent (gland types sensu [25]); (6) number of gill pairs; (7) third lower labial ridge: present, absent; (8) ventral gap in oral disc: definitive, transient, absent; (9) ventrolateral gaps in oral disc: definitive, transient, absent. The developmental sequences were analyzed with R-package Pgi2 using Parsimov as cost function [49–50] and performed under the R 3.4 language [51]. As in the sequence heterochrony plots, a shortened sequence (27 events) was used. Intraspecific variable events (33, 34), events with low changes in the order (2, 4), and active feeding (27) were excluded, and first marginal papillae and marginal papillae with larval configuration were used as descriptors of marginal papillae ontogeny. Since the absence of an event does not impede ancestry reconstruction, the differentiation of a third gill pair, a third lower labial ridge, and the division of adhesive glands were included in these sequences. The analysis was run for 22 leiuperine species plus 7 species of *Leptodactylus* (previously analyzed by [37]) and *Odontophrynus americanus* as the outgroup. We performed 26 independent runs with 200 cycles each one (and 200 replications per cycles), and a simple pseudo-consensus (R script in S2 Appendix). Unlike the plots of sequence heterochrony that emphasize the direct comparative analysis between species, the optimization of the developmental sequences on a phylogenetic hypothesis allows to reconstruct ancestral trajectories and to identify heterochronic shifts in an evolutionary approach.

## Results and discussion

**Fig 1:**
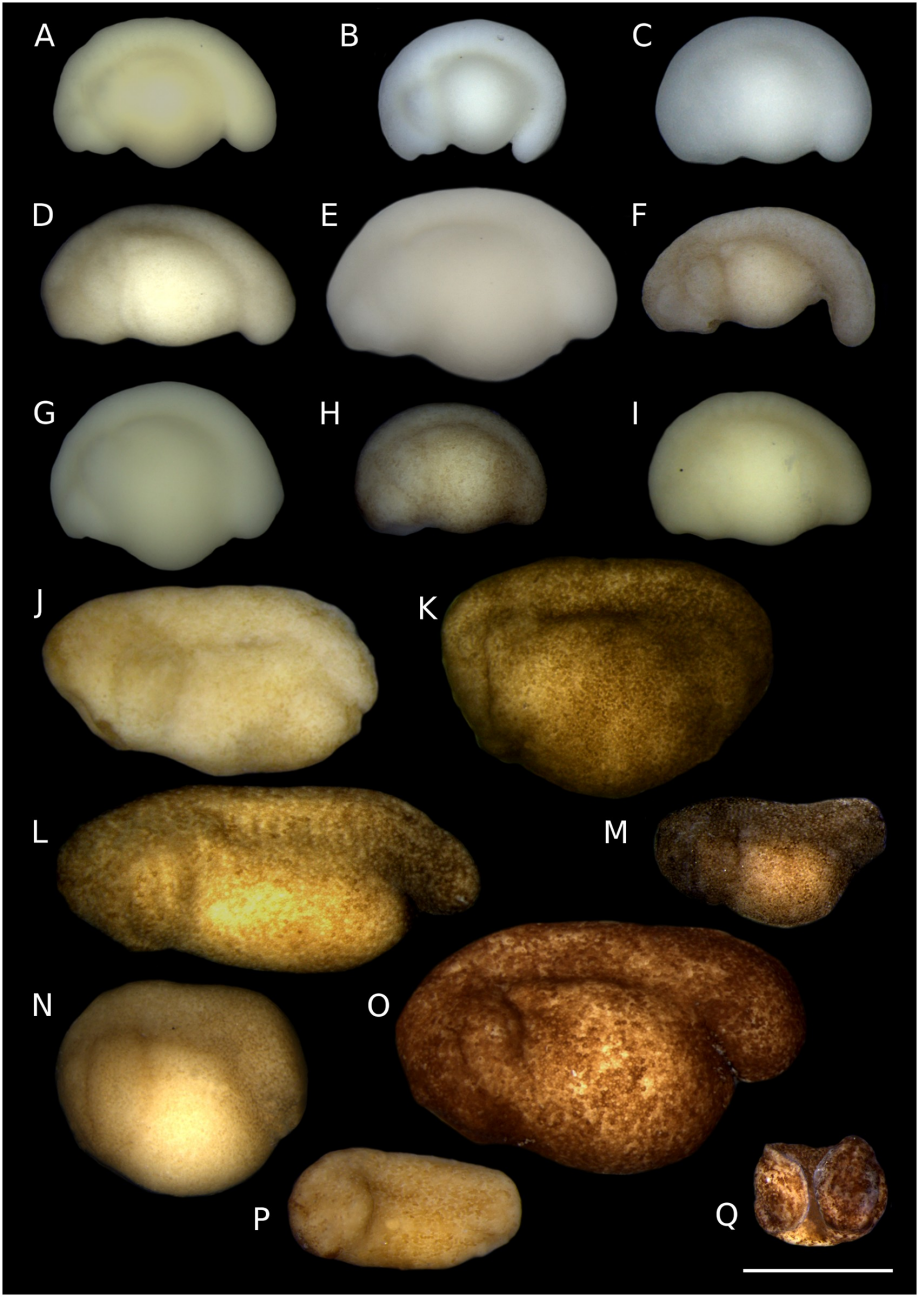
Leiuperinae embryos at tailbud stage. *Physalaemus*: (A) *Ph*. aff. *albonotatus*. (B) *Ph*. *albifrons*. (C) *Ph*. *albonotatus*. (D) *Ph*. *biligonigerus*. (E) *Ph*. *carrizorum*. (F) *Ph*. *cicada*. (G) *Ph*. *cuvieri*. (H) *Ph*. *riograndensis*. (I) *Ph*. *santafecinus*. *Pleurodema*: (J) *Pl*. *borellii*. (K) *Pl*. *bufoninum*. (L) *Pl*. *cordobae*. (M) *Pl*. *diplolister*. (N) *Pl*. *guayapae*. (O) *Pl*. *thaul*. *Pseudopaludicola*: (P) *Ps*. *falcipes*. (Q) *Ps*. *mystacalis*. Scale bar 1 mm. Five species were not included because tailbud embryos were lacking in our ontogenetic series. Note the diversity of size, shape, and pigmentation, from small, kyphotic, white embryos of most *Physalaemus*, to large, almost straight, dark embryos in some *Pleurodema*.

**Fig 2:**
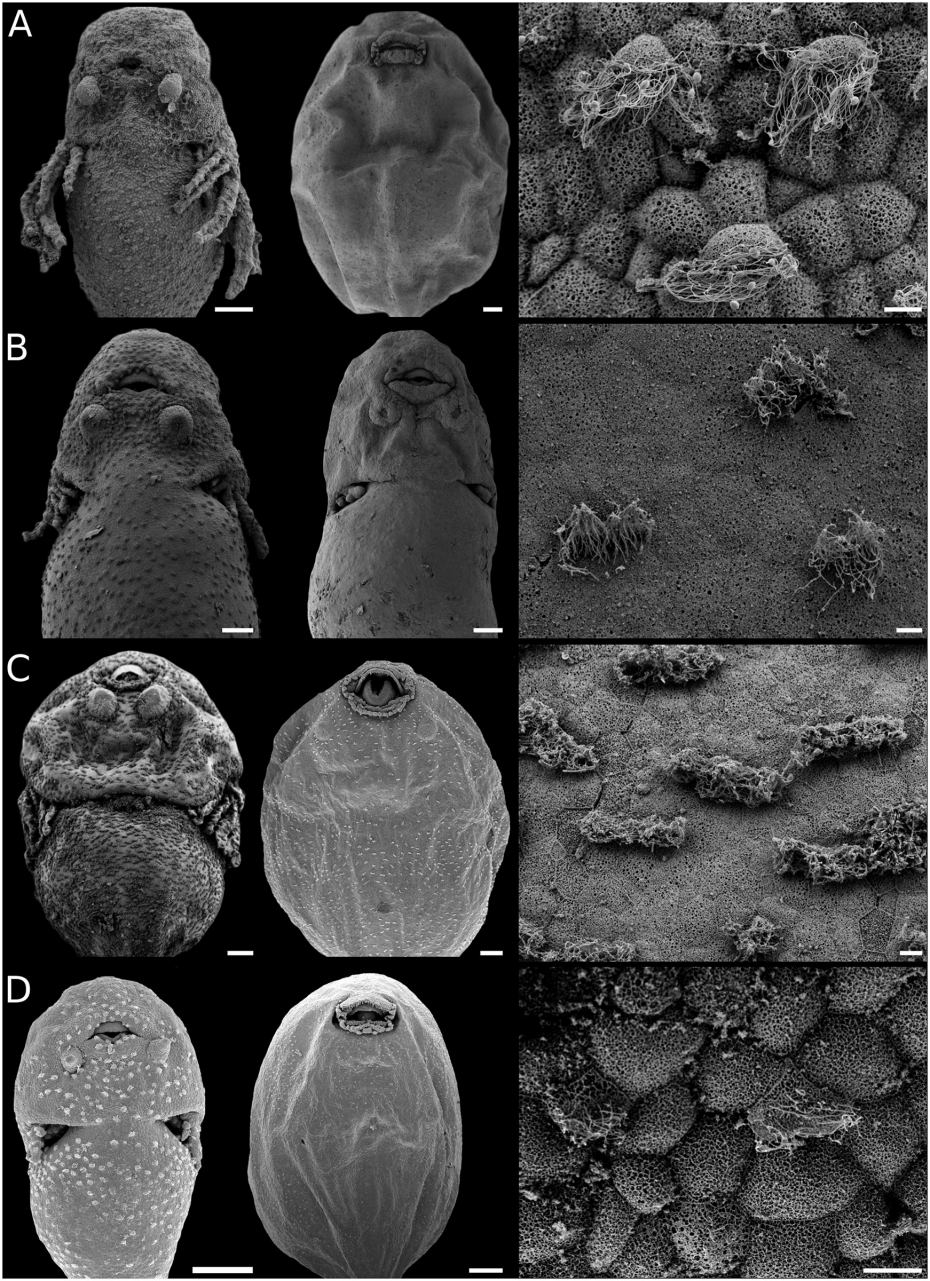
Body ciliation in leiuperine embryos. Ventral views of specimens with fully developed ciliation (left), older embryos with ciliation highly reduced (middle), and details of ciliated cells (sampled from the abdominal region at full development; right). (A) *Physalaemus* aff. *albonotatus*, embryos at GS23 and GS25. (B) *Pleurodema thaul*, embryos at GS22 and GS23. (C) *Pl*. *nebulosum* clade: *Pl*. *nebulosum*, embryo at GS23, and *Pl*. *guayapae*, embryo at GS27. (D) *Pseudopaludicola*: *Ps*. *falcipes*, embryos at GS23 and GS25, and detail of ciliated cells of *Ps*. *mystacalis*. Scale bars 200μm (left and middle), 10μm (right). Note the rounded or oblong cells, and the differences in regression timing among *Pleurodema* species, early in *Pl*. *thaul* and highly delayed in *Pl*. *guayapae*.

**Fig 3:**
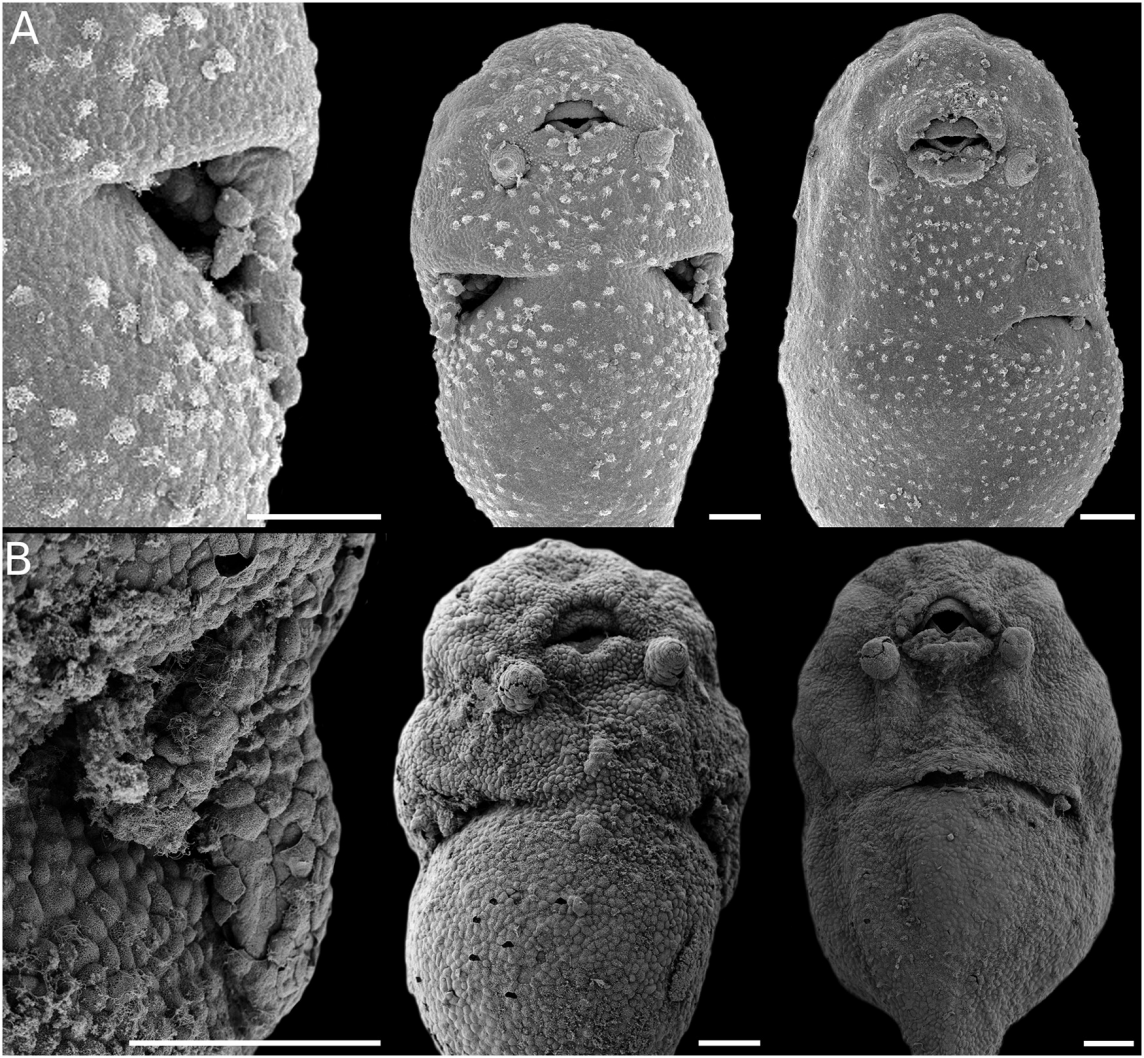
Gill development in *Pseudopaludicola*. Details of gill morphology and ciliation (left), embryos with gills at full development (middle), and gills regressing (right). (A) *Ps*. *falcipes*. (B) *Ps*. *mystacalis*. Scale bars 100μm. Note the overall poor development in the genus.

**Fig 4:**
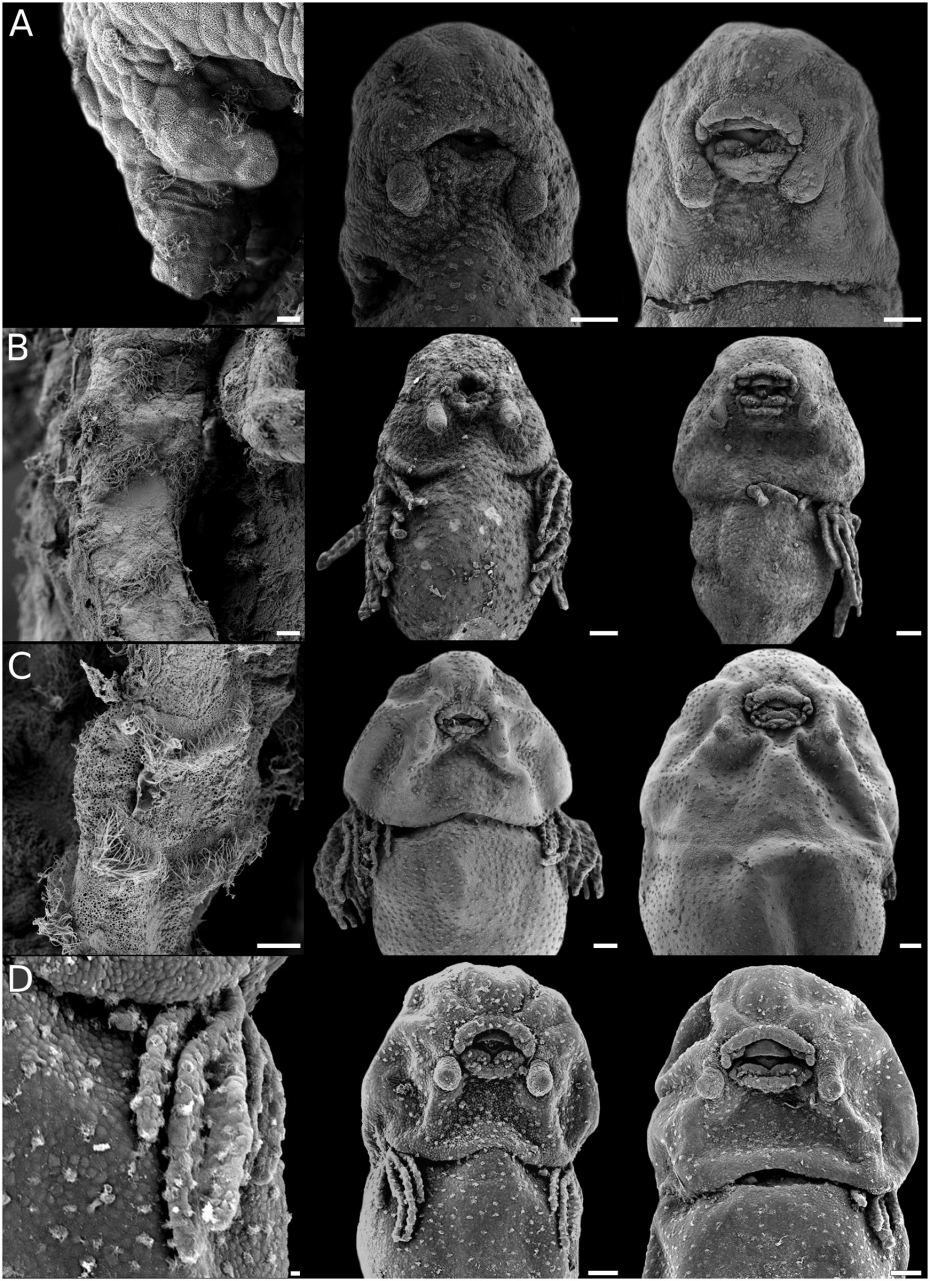
Gill development in *Physalaemus*. Details of gill morphology and ciliation (left), embryos with gills at full development (middle), and gills regressing (right). (A) *Ph*. *fernandezae*. (B) *Ph*. *albifrons*. (C) *Ph*. *carrizorum*. (D) *Ph*. *riograndensis*. Scale bars 10μm (left), 100μm (middle and right). Note the poor and brief development of gills of *Ph*. *fernandezae* as compared with the other species in the genus.

**Fig 5:**
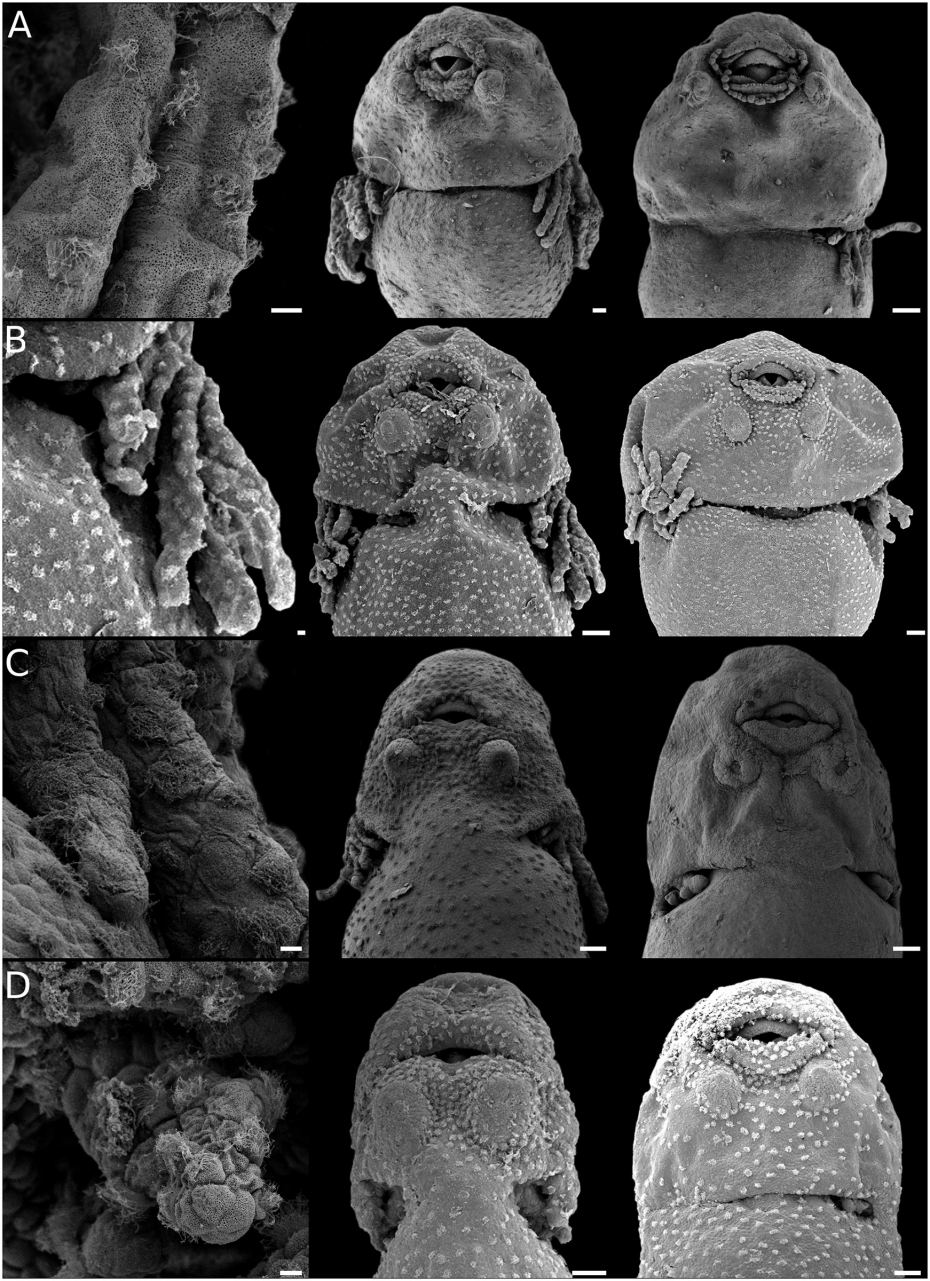
Gill development in *Pleurodema*. Details of gill morphology and ciliation (left), embryos with gills at full development (middle), and gills regressing (right). (A) *Pl*. *diplolister*. (B) *Pl*. *guayapae*. (C) *Pl*. *thaul*. (D) *Pl*. *bibroni*. Scale bar 10μm (left), 100μm (middle and right). Note the comparatively poor and brief development of gills in *Pl*. *thaul* and *Pl*. *bibroni*.

**Fig 6:**
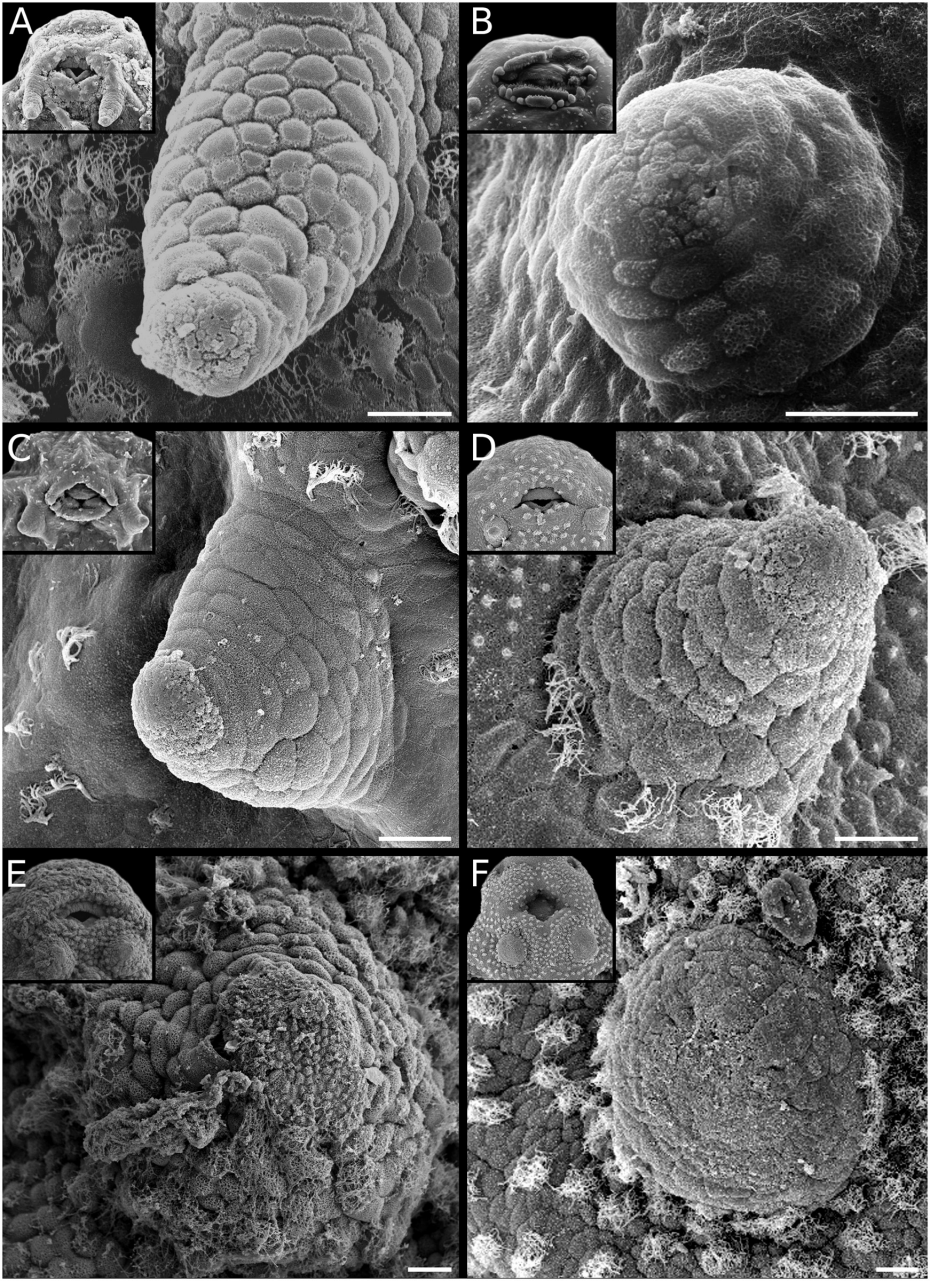
Diversity of adhesive glands in Leiuperinae. *Physalaemus*: (A) *Ph*. *albonotatus*. (B) *Ph*. *henselii*. (C) *Ph*. *santafecinus*. (D) *Pseudopaludicola falcipes*. *Pleurodema*: (E) *Pl*. *bibroni*. (F) *Pl*. *guayapae*. Scale bars 20μm. Note the general arrangement in the insets, and the differences in size and shape of *Pleurodema* glands as compared with those of *Physalaemus* and *Pseudopaludicola*.

**Fig 7:**
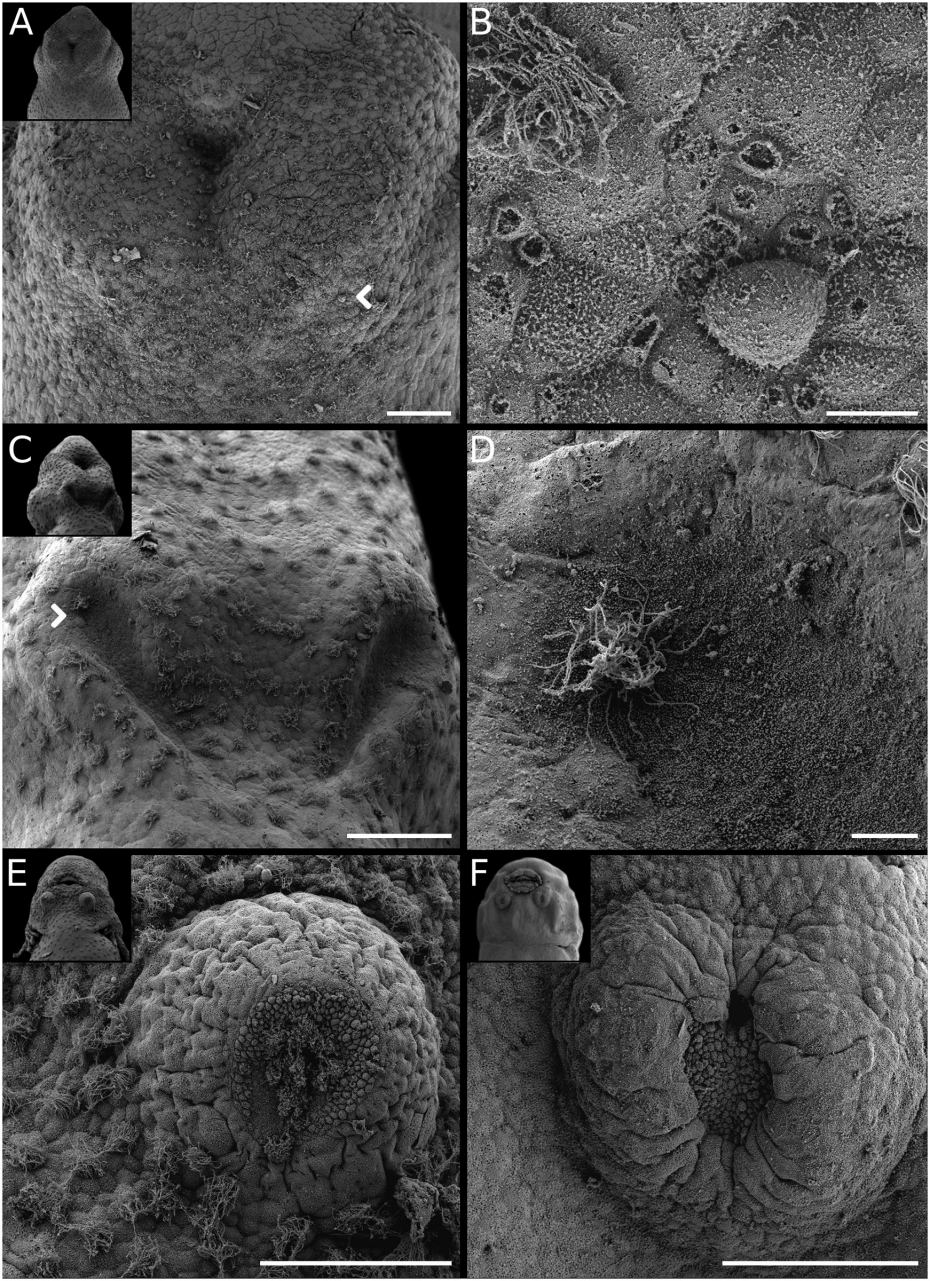
Adhesive gland ontogeny in *Pleurodema thaul*. (A) Initial morphogenetic field at GS17. (B) Detail of cells with microvilli at the secretory region (arrow in A). (C) Morphogenetic field split and in a V-shape configuration at GS19. (D) Detail of secretory region (arrow in C). (E) Adhesive gland at full development–GS23–, as two circular structures with central secretory tissue. (F) Regressing adhesive glands at GS24–25, with a reduced secretory region. Scale bars 100μm, excepting B and D 10μm. In the insets, besides the differences in gland morphology note the position ventral to the oral disc conserved along the ontogeny.

**Fig 8:**
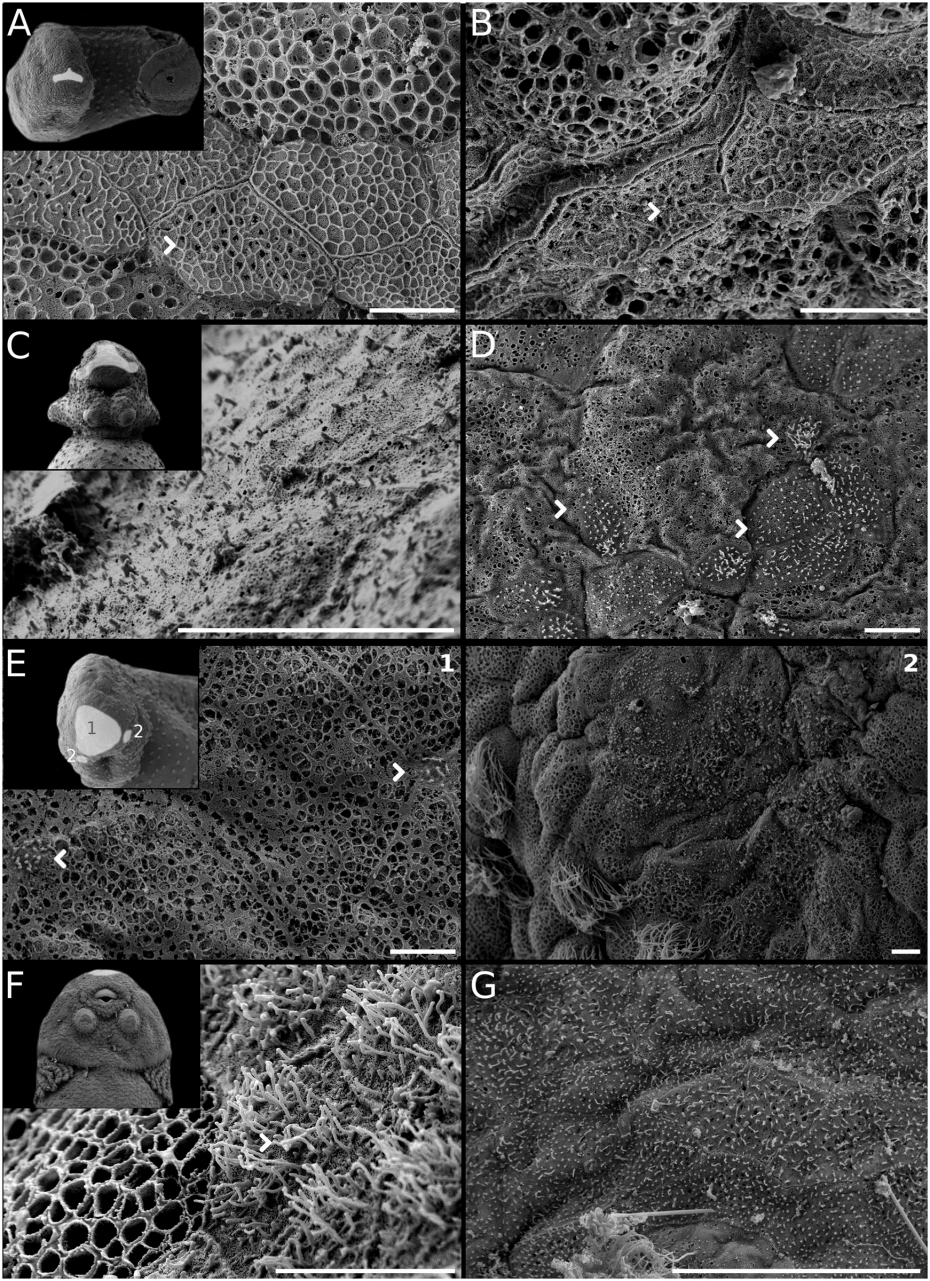
Diversity of hatching glands in Leiuperinae. *Pseudopaludicola*: (A) *Ps*. *falcipes* at GS17–18. (B) *Ps*. *mystacalis* at GS17–18. *Physalaemus*: (C) *Ph*. *albifrons* at GS17–18. (D) *Ph*. *biligonigerus* at GS17–18. (E) *Ph*. *fernandezae* at GS17–18. *Pleurodema*: (F) *Pl*. *borellii* at GS22–23. (G) *Pl*. *bibroni* at GS17–18. Scale bars 5μm. Individual secretory cells are indicated with arrows. Note the general arrangement as shaded areas in the insets, and the long microvilli in *Pl*. *borellii*.

**Fig 9:**
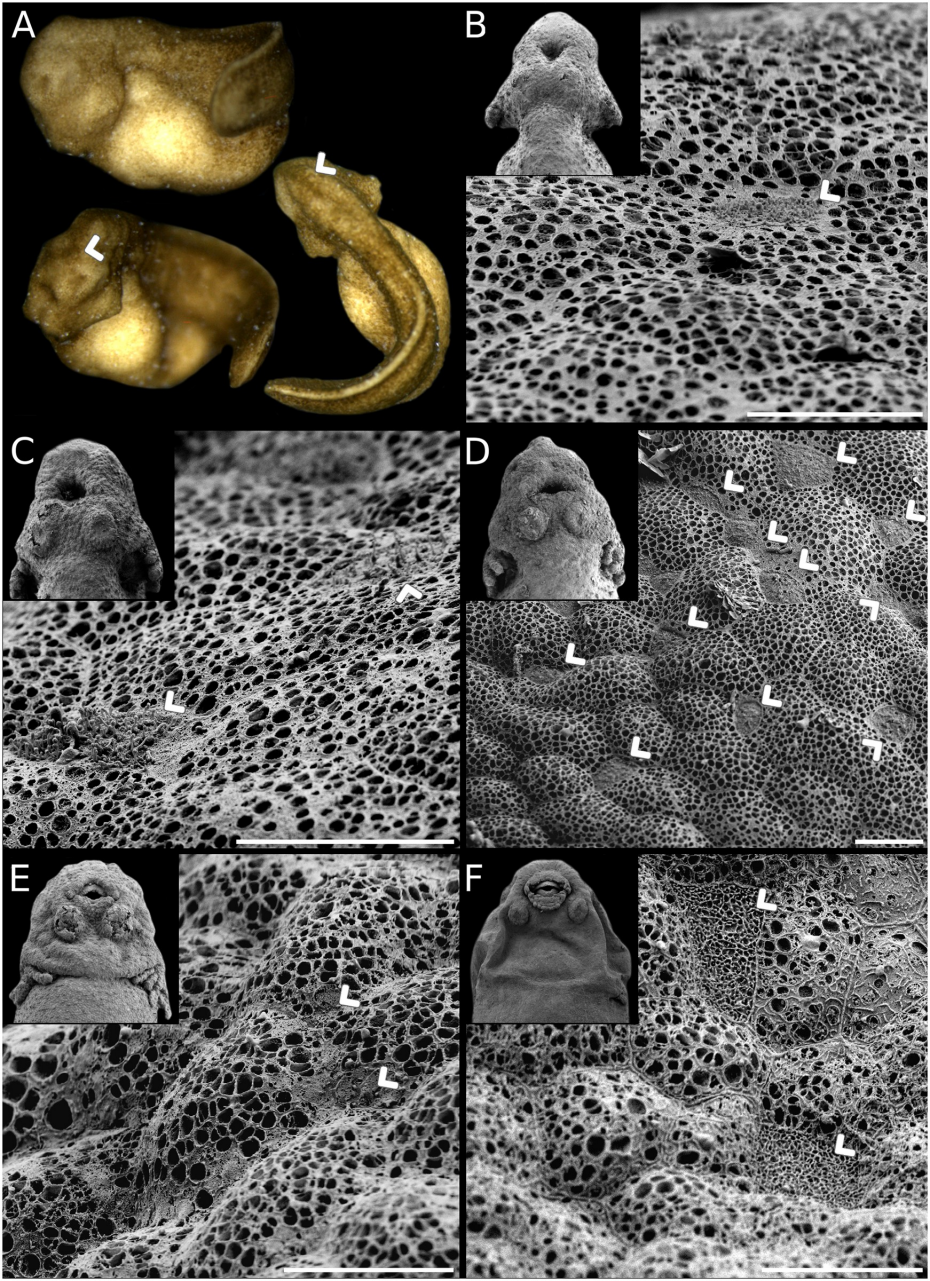
Hatching gland ontogeny in *Pleurodema bufoninum*. (A) Embryo at GS19, showing gland arrangement and pigmentation in the cephalic region. Secretory cells (arrows) in embryos at (B) GS20. (C) GS21. (D) GS22. (E) GS23. (F) GS25. Scale bars 10μm. The densest arrangement take place around GS22, co-occurring with gills at full development.

**Fig 10:**
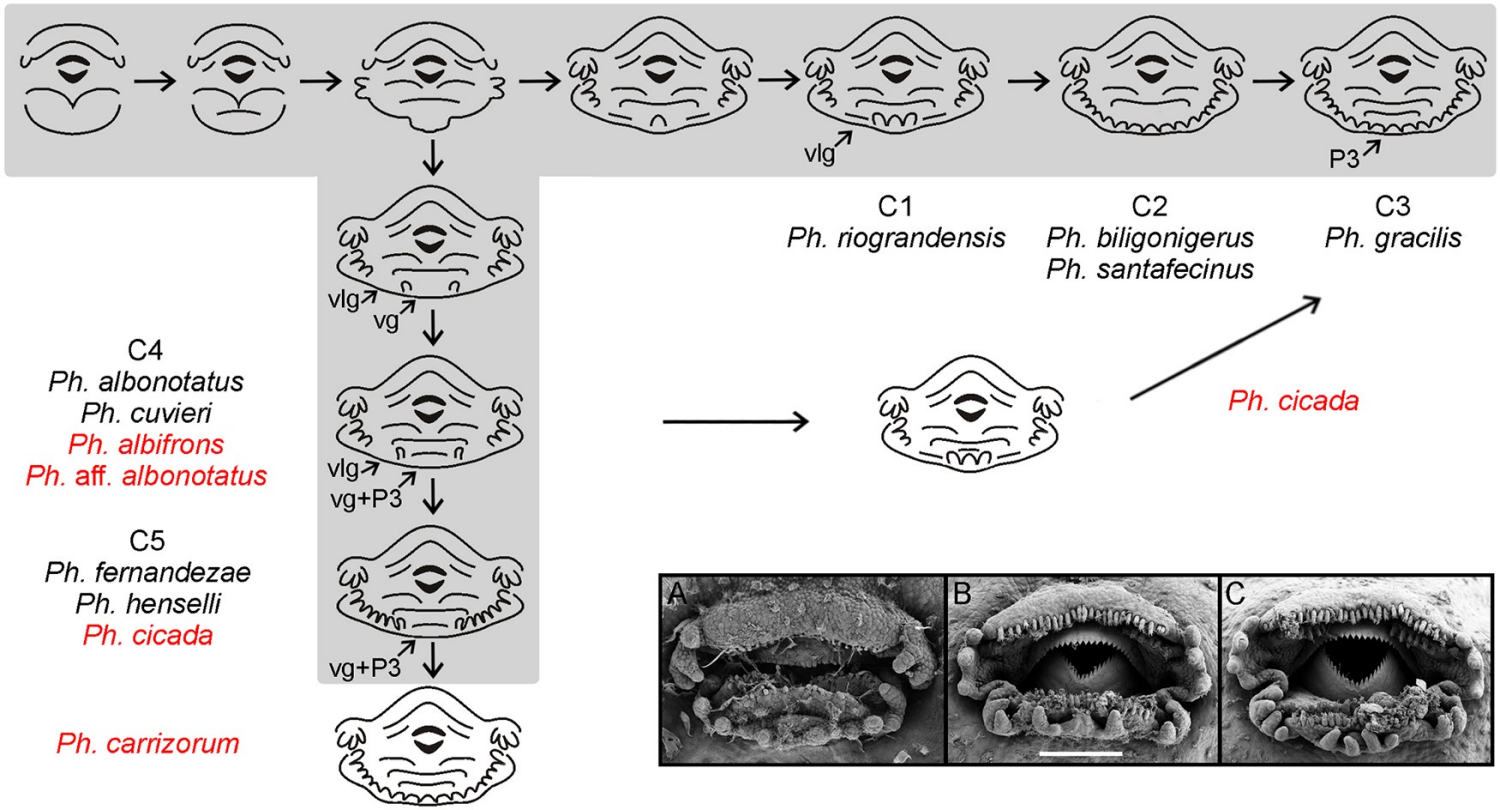
Scheme of oral disc trajectories in *Physalaemus*. Shaded trajectories and assignation of species to oral configurations labeled C1-C5 are as modeled in Vera Candioti et al. [35]. Additional species in our work are indicated in red and their larval oral configurations and trajectories inserted on the original scheme. The diagram emphasizes on ventral and ventrolateral gaps development (VG and VLG respectively), whereas the timing of differentiation of row P3 regarding mental papillae is simplified since it can vary between species sharing the same trajectory. Micrographs show transient oral discs of (A) *Ph*. *carrizorum*, and (B,C) *Ph*. *cicada*, highlighting representatives of the new trajectories. Note the small papilla filling the ventral gap in *Ph*. *carrizorum*, and the alternative transient morphologies of *Ph*. *cicada* embryos, with or without ventral gap.

**Fig 11:**
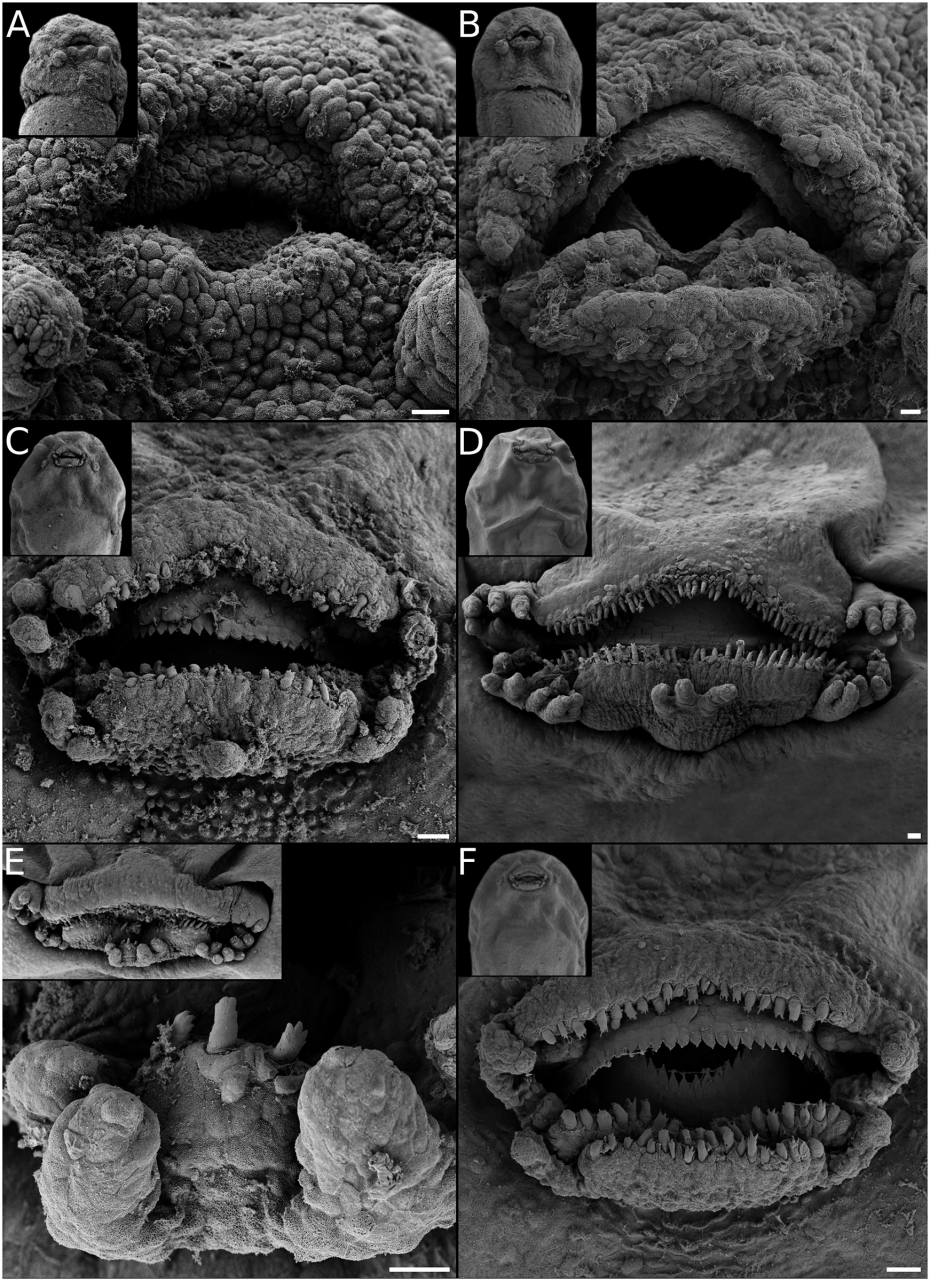
Oral disc ontogeny in *Pseudopaludicola mystacalis*. (A) Rows A1 and P1. (B) Rows A2, P2, and commissural papillae differentiated. (C) Labial teeth on all tooth ridges, marginal papillae at commissures and medially at the mental region. (D) Larval oral disc ending this trajectory, i.e., two lower labial ridges and persisting ventrolateral gaps (oral disc C1 *sensu* [29]). (E) A rare larval oral disc with a third labial tooth row formed from mental papillae/ridge. (F) A further alternative larval oral disc with two lower labial tooth rows and a wide, bufonid-like ventral gap. Scale bars 20μm. Note the highly polymorphic larval oral disc, and the intraspecific diversity in oral developmental pathways they imply.

**Fig 12:**
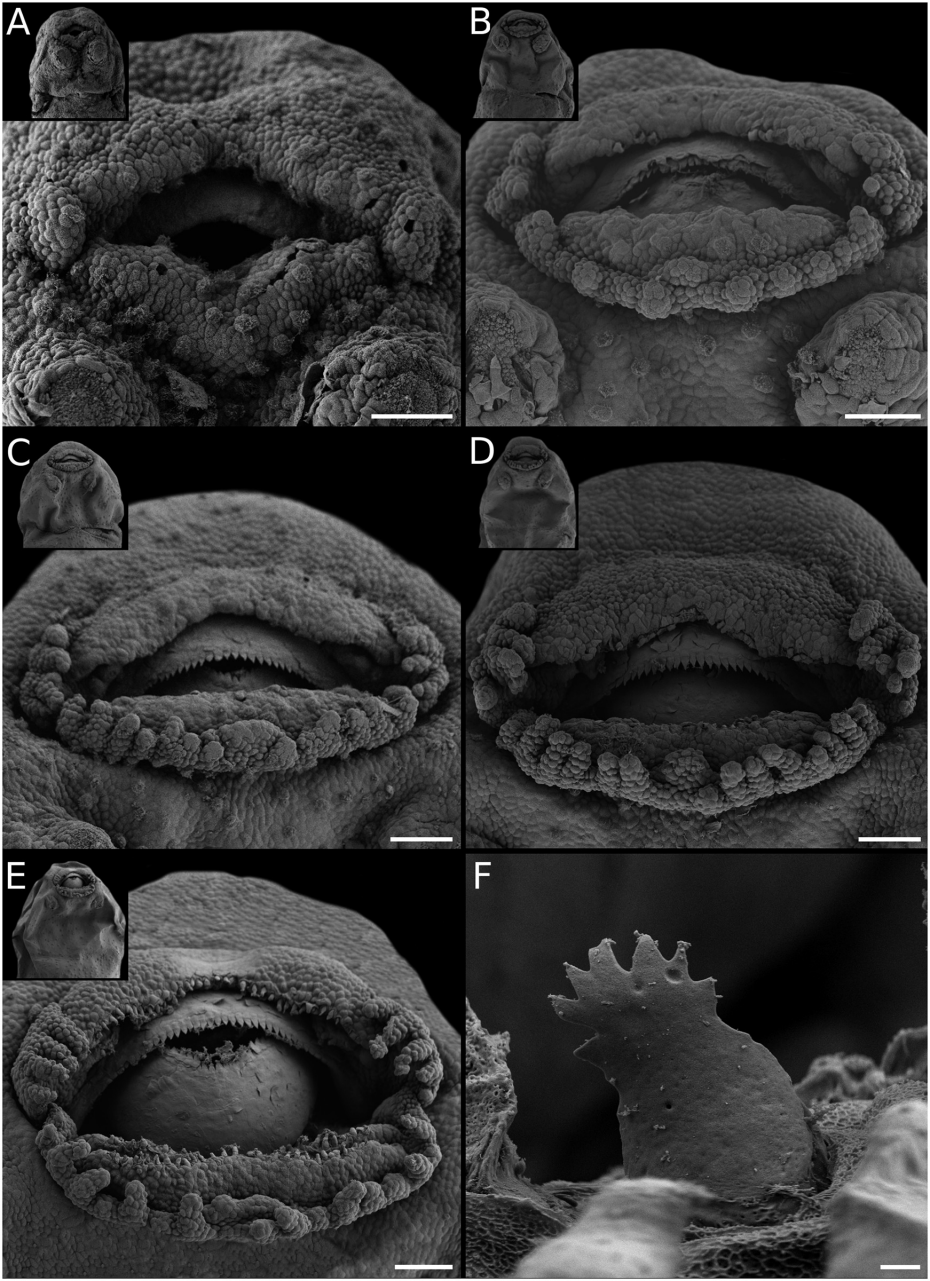
Oral disc ontogeny in *Pleurodema cordobae*. (A) Rows A1, P2, and commissural papillae. (B) Rows A2, P1, and a short P3 are formed, and marginal papillae progress medially. (C) Marginal papillae with ventral gap. (D) Labial teeth emerging on A1, P1, and P2. (E) Labial teeth on A2, and marginal papillae widely spaced. (F) Detail of a labial tooth from P2. Scale bars 20μm, excepting F 2μm. Note the small, transient ventral gap co-occurring with a short P3.

**Fig 13:**
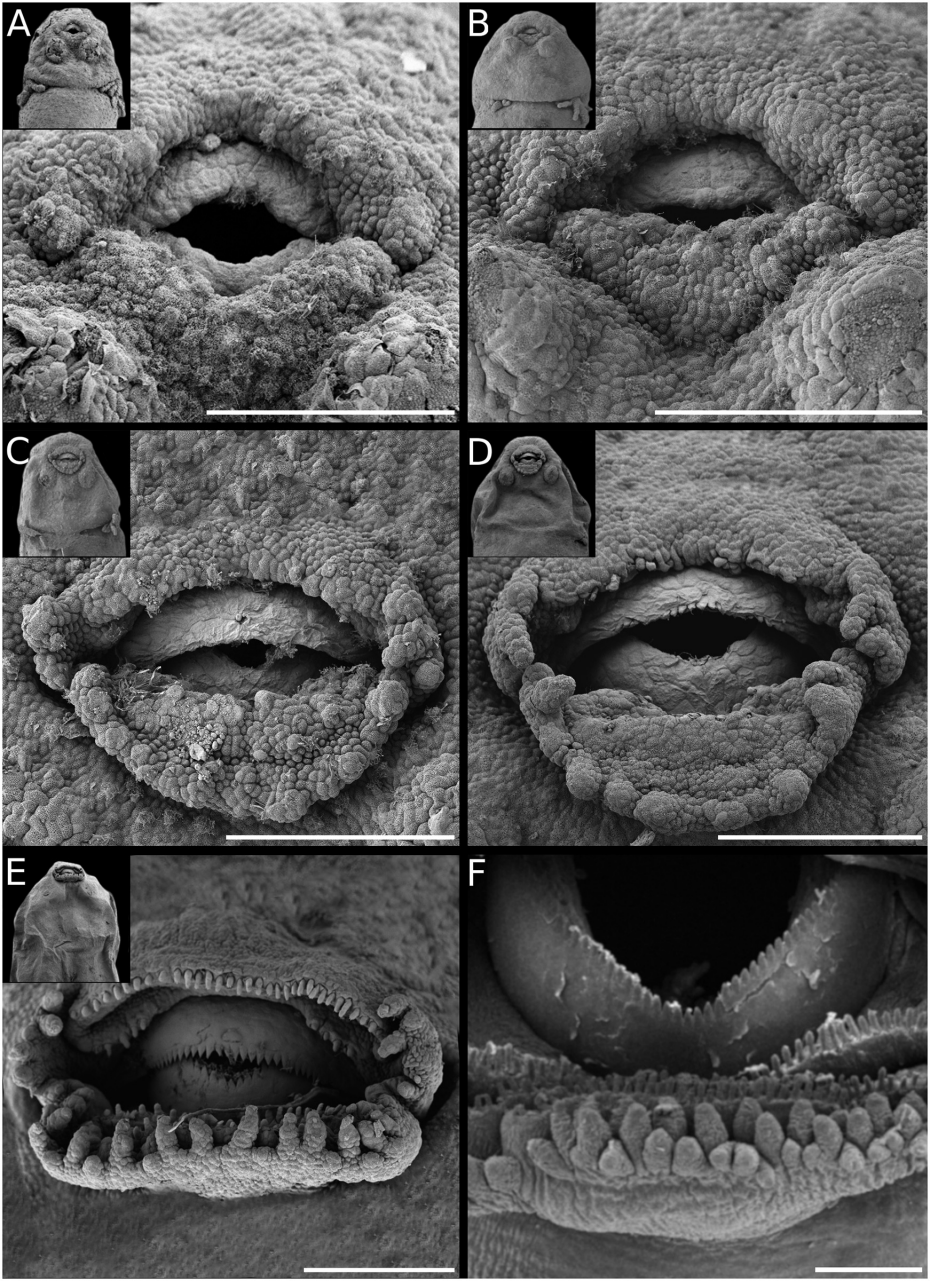
Oral disc ontogeny in the *Pleurodema thaul* clade. *Pl*. *bufoninum*: (A) Rows A1, P2, and commissural papillae evident. (B) Lower lip more defined. (C) P1 and A2 outlined, and lower marginal papillae progressing medially; D) P3 outlined and labial teeth emerging on A1 and P2. (E) Labial teeth on all rows, and marginal papillae widely spaced. *P*. *thaul*: (F) Detail of the lower lip of a Stage 37 tadpole. Scale bars 200μm. Note the larval oral disc with the alternate papillae giving a double-like appearance to the lower lip margin.

**Fig 14:**
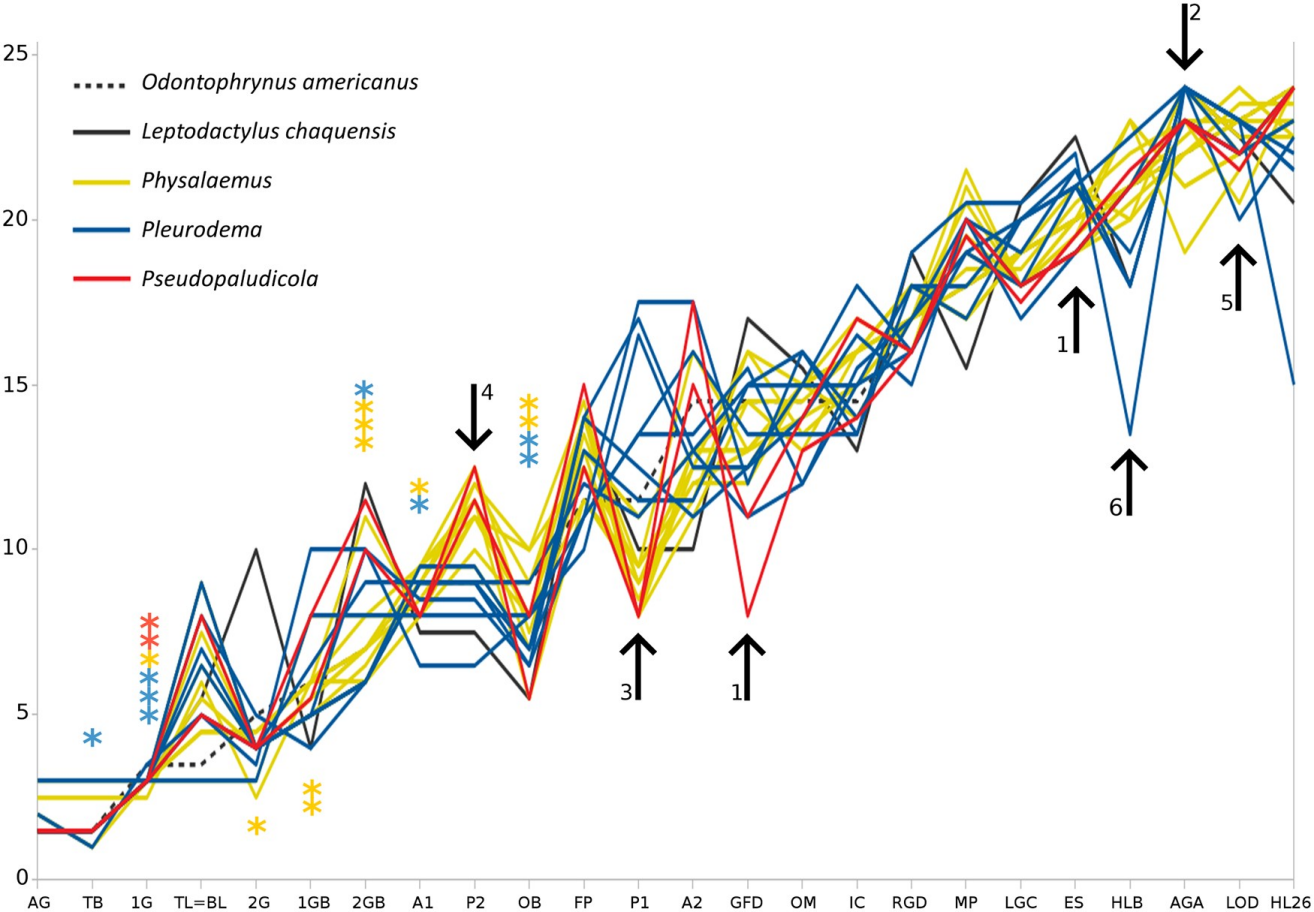
Sequence heterochronies in developmental trajectories of leiuperines. Developmental events (N = 24; data from S3 Appendix; X-axis) are plotted according to their order in the sequence (Y-axis), as compared to the reference trajectory of *Odontophrynus americanus*. AG adhesive gland first visible, TB tail bud, 1G first gill pair bud, TL = BL tail length / body length = 1, 2G second gill pair bud, 1GB first gill pair branched, 2GB second gill pair branched, A1 labial tooth ridge A1, P2 labial tooth ridge P2, OB operculum at gill base, FP first marginal papillae, P1 labial tooth ridge P1, A2 labial tooth ridge A2, GFD gills at full development, OM operculum medially fused, IC first coil in digestive tract, RGC right gill covered by operculum, MP marginal papillae complete, LGC left gill covered by operculum, ES spiracle developed, HLB hind limb buds, AGA adhesive glands absent, LOD oral disc fully formed, HL26 hind limbs at GS26. Species are not individualized since main variations among genera are emphasized. Asterisks point out the event after which hatching occurs for each species. The numbered arrows indicate main heterochronic changes: 1) the accelerated full development of gills and spiracle differentiation of *Pseudopaludicola*; 2) a late adhesive gland regression in *Pleurodema*; 3 and 4) variations in the ontogeny of rows P1 and P2 that differentiate *Pseudopaludicola* and *Physalaemus* from *Pleurodema*; 5) the early acquisition of larval oral disc in *Ph*. *carrizorum*; and 6) the extremely early development of hind limbs in *Pl*. *guayapae*.

**Fig 15:**
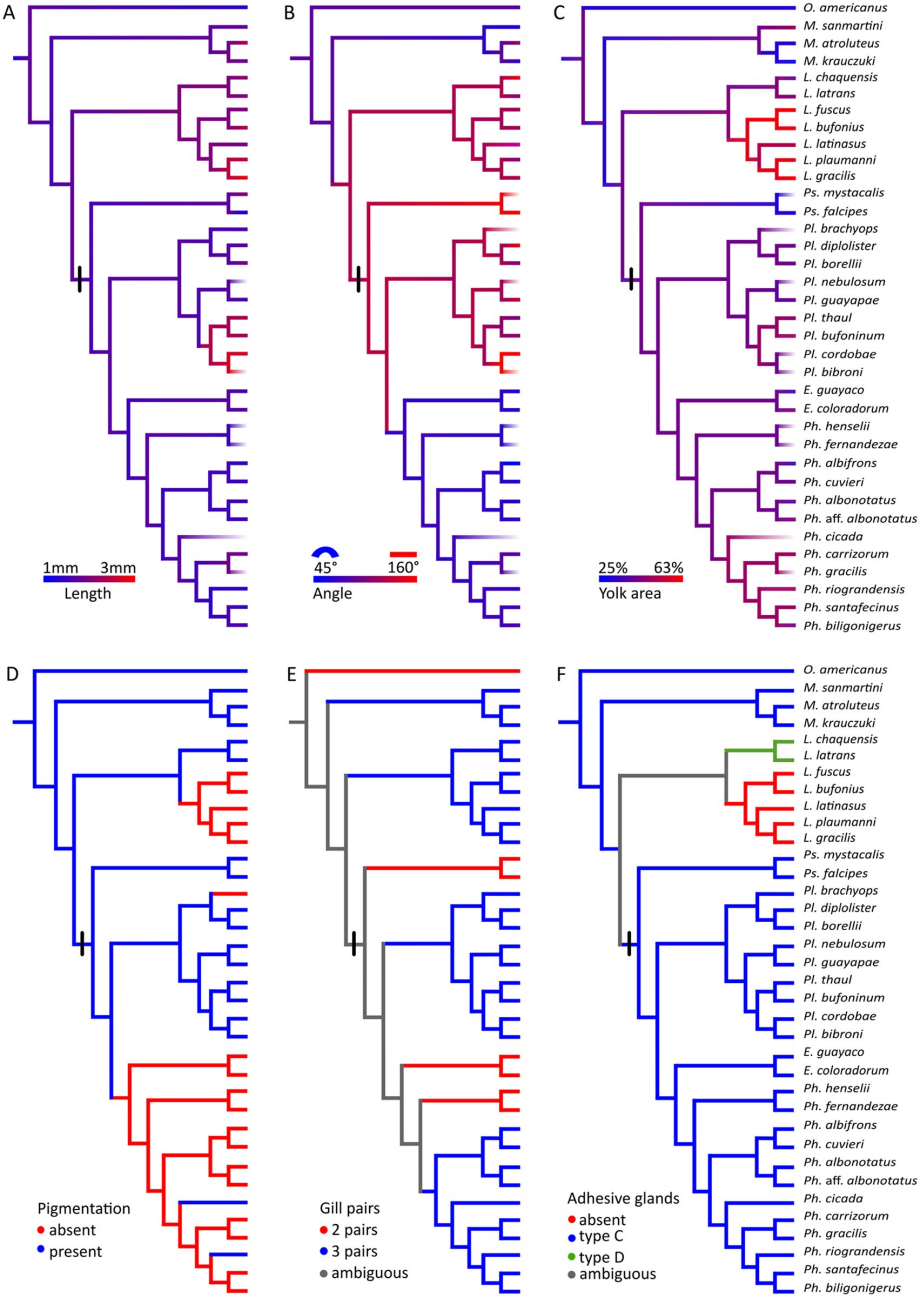
Ancestral reconstruction of embryonic morphological characters. Data are taken from S4 Appendix. The first four characters are recorded in embryos at tailbud stage. (A) Length. (B) Angle of dorsal curvature; the smallest angles correspond to highly kyphotic embryos. (C) Yolk relative area. (D) Pigmentation: absent, present. (E) Gill pairs: 2, 3. (F) Adhesive glands: absent, type C, type D. The black bar indicates the ancestor of Leiuperinae. Note the overall high diversity in leiuperine tailbud embryos, contrasting with comparatively conserved gill and adhesive gland morphologies.

**Fig 16:**
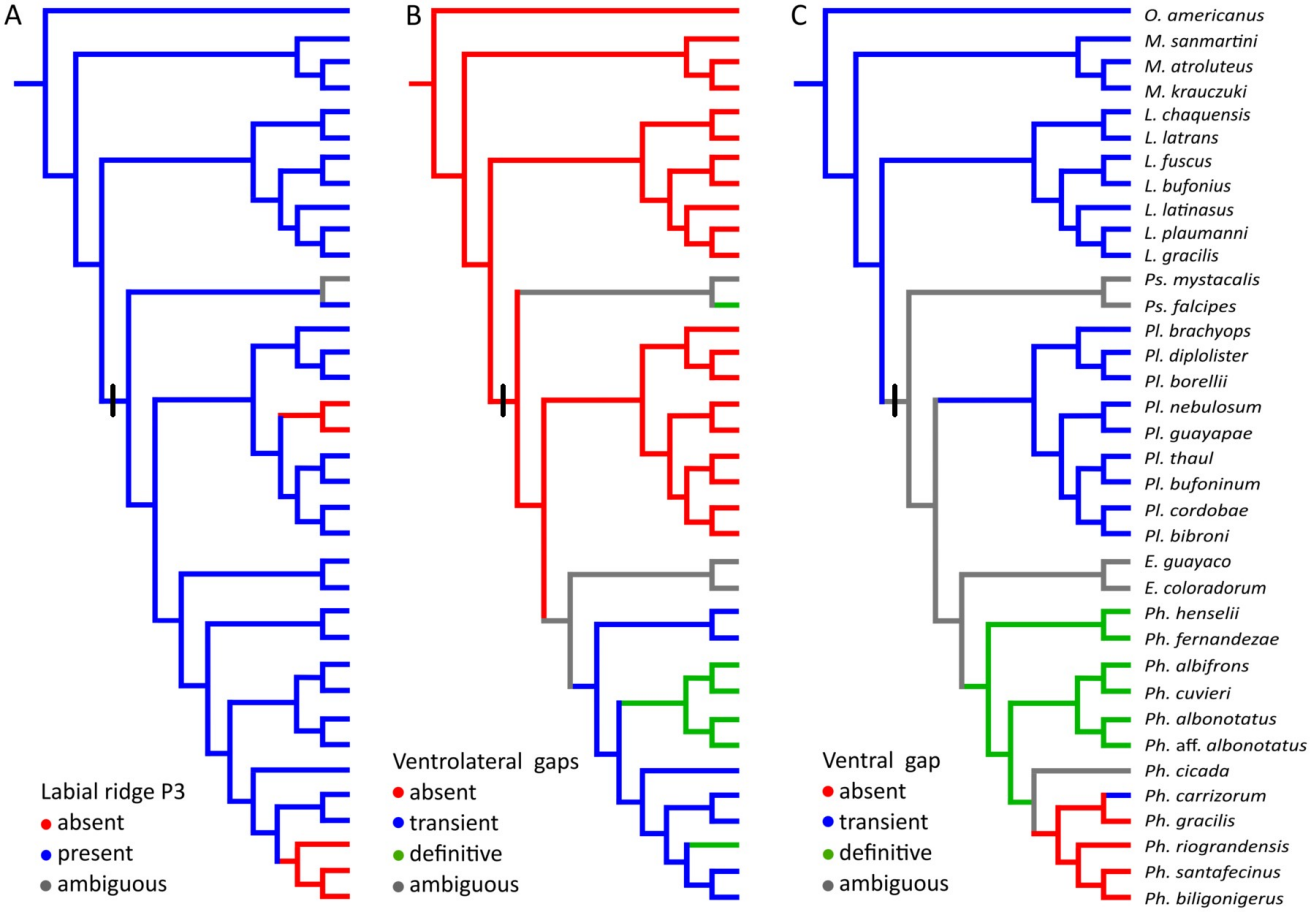
Ancestral reconstruction of oral disc features. Data are taken from S4 Appendix. (A) Labial tooth row P3. (B) Ventrolateral gaps. (C) Ventral gap. The black bar indicates the ancestor of Leiuperinae. Ambiguity in species we studied indicates the intraspecific occurrence of at least two alternative states. Developmental data on *Engystomops* are lacking to ascertain whether gaps are absent or transient, so ambiguity is indicated as well. Note the overall highly diverse oral features in leiuperines, including two or three lower labial tooth rows combined with a variety of marginal papillae arrangements.

**Fig 17:**
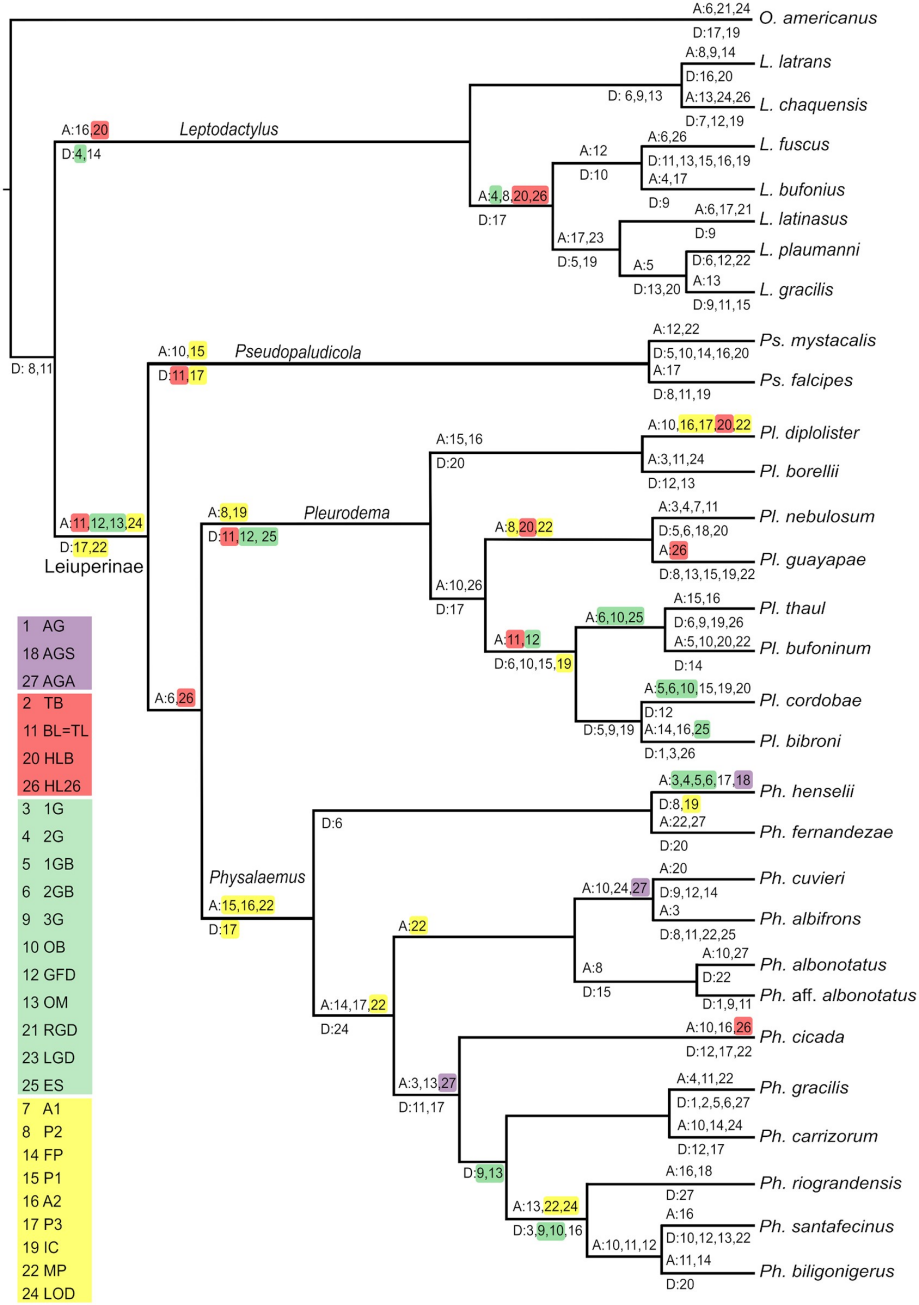
Ancestral reconstruction of developmental sequences. Data are taken from S5 Appendix. Events (N = 27): (1) AG adhesive gland first visible, (2) TB tail bud, (3) 1G first gill pair bud, (4) 2G second gill pair bud, (5) 1GB first gill pair branched, (6) 2GB second gill pair branched, (7) A1 labial tooth ridge A1, (8) P2 labial tooth ridge P2, (9) 3G third gill pair bud, (10) OB operculum at gill base, (11) TL = BL tail length / body length = 1, (12) GFD gills at full development, (13) OM operculum medially fused, (14) FP first marginal papillae, (15) P1 labial tooth ridge P1, (16) A2 labial tooth ridge A2, (17) P3 labial tooth ridge P3, (18) AGS adhesive glands separated, (19) IC first coil in the digestive tract (20) HLB hind limb buds, (21) RGC right gill covered by operculum, (22) MP marginal papillae complete, (23) LGC left gill covered by operculum, (24) LOD oral disc fully formed (25) ES spiracle developed, (26) HL26 hind limbs at GS26, (27) AGA adhesive glands absent. Accelerations (A) and decelerations (D) of events reconstructed as synapomorphies are indicated above and below branches respectively, and main shifts discussed in the text are colored according to morphological structures they describe: adhesive glands (violet), tail and hind limbs (red), gills (green), and oral disc (yellow).

The evidence presented in this work plus the previous information available from the literature review show that the main differences during the early development of leiuperines involve the embryo morphology at tailbud stage, the external gills, the hatching gland, and the oral disc. Results obtained for each character are illustrated and discussed in the next section (Interspecific variation in embryonic characters of Leiuperinae; Figs 1–13). Detailed information of each species is consigned as supplementary information, including morphological aspects, measurements (S1 Table), sequences of ontogenetic events (S2 Table), and matrices for sequence heterochrony analysis (S3 Appendix) and ancestral reconstructions (S4 and S5 Appendices). These data are summarized and presented comparatively in a sequence heterochrony plot (Fig 14), and in the ancestral state reconstruction of morphological (Figs 15 and 16) and developmental event (Fig 17) data. The evolution of developmental trajectories is addressed in the final section (Evolution of early ontogenetic trajectories in Leiuperinae), emphasizing on heterochronic shifts in major clades. Ecomorphological considerations related to oviposition and embryo habitat are also summarized in this last section.

### Interspecific variation in embryonic characters of Leiuperinae

#### Morphology at tailbud stage

At tailbud stage (GS17–18), leiuperine embryos vary widely in size, shape, and pigmentation (Figs 1 and 15A–15D). Body length is highly variable, with some species being twice as long as others (S2 Table). Embryos of *Pleurodema* have an average body length of 2.06 mm (1.65–2.70 mm). Transformation to a larger body is recovered in the clade joining the *Pl*. *thaul* and *Pl*. *bibroni* groups (mean length = 2.47 mm), which have the largest embryos for leiuperines. The clade *Physalaemus* + *Engystomops* has the smallest embryos, with mean body sizes of 1.55 mm (1.36–1.90 mm). Embryos of *Pseudopaludicola* significantly differ; *Ps*. *falcipes* tailbud embryos are only 1.06 mm long whereas embryos of *Ps*. *mystacalis* are two times longer, reaching 2.10 mm. Embryos of the confamilial genus *Leptodactylus* show a comparable range of body length, with lower values in species of the *L*. *latrans* group and larger ones in the *L*. *fuscus* group [37].

Leiuperinae embryos at tailbud stage differ also in dorsal curvature and yolk shape. *Engystomops* and *Physalaemus* embryos are typically curved lying upon a subspherical yolk, while *Pleurodema* and *Pseudopaludicola* embryos are straighter and have an oblong yolk (Fig 1). As discussed in a previous work [52], descriptions of dorsal curvature in terms of kyphosis and lordosis are not detailed enough to describe interspecific variation. Furthermore, yolk shape and area that help to characterize embryo shape should be discussed separately from body curvature. In order to obtain a more precise description of the yolk shape and area, we recorded the body region where the curvature occurs, and measured the angle of body curvature and the relative yolk area, independently reconstructing ancestral states for those two characters (Fig 15B and 15C; S1 Table). The ancestral states for Leiuperinae are slightly kyphotic embryos with dorsal curvatures of 112–120° and yolk areas of about 40% of the body area. These are similar to the plesiomorphic states of Leptodactylidae. A slight curvature is maintained in *Pseudopaludicola* and *Pleurodema—*in this last case mainly in the caudal region with average angles of 119°—with straighter embryos in *Ps*. *falcipes* and *Pl*. *cordobae*. Yolk area is slightly larger in the *Pl*. *thaul* clade. In *Engystomops* + *Physalaemus* the highly pronounced kyphosis along the whole body (64–71°) and the rounded yolk render embryos almost spherical. The yolk area increases significantly only in the derived clade of *Ph*. *cicada + Ph*. *santafecinus*. In contrast, *Leptodactylus* has large, highly curved, and yolked (≥ 50%) embryos in the *L*. *fuscus* group, whereas embryos in the *L*. *latrans* group almost lack dorsal curvature and their yolk area represents about 40% of their body area [37].

Regarding coloration, pigmented embryos are the plesiomorphic state of Leiuperinae, with two independent transformations to whitish embryos in *Pl*. *brachyops* [32] and the clade joining *Engystomops* + *Physalaemus* (Fig 15D). Pigmentation in *Pseudopaludicola* and *Pleurodema* embryos range from light brown in *Pl*. *borellii* to dark brown in *Ps*. *mystacalis* and the *Pl*. *thaul* clade. In *Engystomops* and most *Physalaemus* embryos are initially whitish and scattered dots appear anterodorsally once the second gill pair develops. Two reversals are observed in the pigmented embryos of *Ph*. *cicada* and *Ph*. *riograndensis*. The lack of pigmentation has been reported for numerous anuran embryos, and within Leptodactylidae it was described in species of the *Leptodactylus fuscus* group [37] and suspected from egg features in *Edalorhina* (the sister genus of *Engystomops*), species of the *Ph*. *signifer* clade, and *Adenomera* (e.g., [53–55]).

The relationship among size, shape, and pigmentation is well documented in several groups, and highly kyphotic embryos are often also large, yolky, and unpigmented (e.g., [37, 56]). Developmental bases for the correlation between yolk amount and pigmentation extent have been suggested [56], but the relationships between the type of cleavage, yolk uptake, pigment production and mobility, and the vast diversity of reproductive modes in anurans is still under discussion [57]. Some other diverging patterns of association between embryo size, shape, and pigmentation have been described. Kyphotic embryos can also be small, like those of most species of *Physalaemus*, *Hyperolius puncticulatus*, *Atelopus*, and several *Melanophryniscus* [52, 58], and pigmented, as in *Ph*. *riograndensis*, some *Melanophryniscus*, and species of *Batrachyla* and *Odontophrynus* [52, 59–60]. Regarding yolk amount, unlike several kyphotic embryos with relative yolk areas larger than 50%, embryos of *Engystomops*, *Physalaemus*, *Melanophryniscus*, and *O*. *americanus* have small yolk provision (30–50%).

#### Body ciliation

Results in this section emphasize some areas of the embryo body (the ventral aspect, gills, cephalic region); distribution of ciliated cells in the body dorsum and tail is undersampled. Patterns of ciliation in Leiuperinae vary in distribution, cell shape, and persistence (Fig 2). The ciliated cells, rounded or polygonal in most species, are more densely arranged in the cephalic region, especially on those areas surrounding the nares, the oral disc, and the adhesive glands. Body ciliation changes during ontogeny, in general with maximum density at full gill development, and a progressive regression post GS25. Embryos of the *Pleurodema thaul* clade and of the *Physalaemus henselii* group have sparse ciliated cells that regress earlier, with gills still exposed (Fig 2B). At the other extreme, embryos of *Pl*. *borellii* and the *Pl*. *nebulosum* clade have large, densely arranged ciliated cells that regress late after hind limb emergence (Fig 2C). Likewise, body ciliation in *Pseudopaludicola falcipes* regresses once hind limbs reach GS27 (Fig 2D). A late regression of body ciliation was also described for *E*. *pustulosus*, and in the leptodactyline *Leptodactylus fuscus* [28].

#### External gills

The external gills in leiuperines develop between GS18 and GS24, and dispose laterally to the body in a configuration similar to most anuran embryos. Main variations occur in the number and length of pairs and filaments (Figs 3–5; S1 Table). The ambiguity in the reconstructed pair number for the subfamily ancestor (Fig 15E) could be solved with further information on embryos of *Edalorhina* and species of the *Physalaemus signifer* clade. Two gill pairs are recovered as a homoplastic feature for *Engystomops* (see also [23, 61]), *Pseudopaludicola* (Fig 3), and the *Ph*. *henselii* group (Fig 4A). All these species have poorly developed gills, with only 3–6 filaments, primary filaments among the shortest in the sample (0.24–0.30 mm; S1 Table), and ciliation relatively scarce. A comparative analysis of developmental sequences (Fig 14, S2 Table) shows that gill ontogeny was also shorter in these species, i.e., maximum gill development and gill regression occurs earlier than in most leiuperines.

Embryos of *Pleurodema* and the clade *Physalaemus cuvieri + Ph*. *biligonigerus* (Fig 15E) develop three gill pairs (see also [16, 34]. Embryos of *Physalaemus* (except in the *Ph*. *henselii* group) and the clades *Pl*. *brachyops* and *Pl*. *nebulosum* show the most complex gills at full development. In these taxa, the first pair branches into 6–12 filaments, primary filaments are long (0.55–1.04 mm), third pair always grows beyond the operculum margin (Figs 4B, 4C, 5A and 5B; S1 Table), and gill ciliation is profuse. In contrast, gills in the clade formed by the *Pl*. *thaul* + *Pl*. *bibroni* groups are scarcely developed and the shortest in *Pleurodema* (Fig 5C and 5D).

The literature reports variations in leiuperine gill structure. For instance, embryos of *Engystomops pustulosus* were described with two poorly developed gill pairs [23] or three long and ciliated gill pairs [27]. Besides an explanation related to cryptic diversity in this widely distributed species (as suggested by advertisement call and allozyme evidence [62]), intraspecific variations in gill structure should be explored in this and other leiuperines. In our study, intraspecific variation is low, but three cases are noteworthy. Embryos of a single clutch of *Pl*. *borellii* presented four gill pairs instead of three, and we found maximum length of gills widely different in embryos of *Physalaemus santafecinus* and *Ph*. *riograndensis* from different clutches. Wider sampling and experimental works are desirable in order to further characterize these polymorphisms.

#### Adhesive glands

The optimization of adhesive gland characters recovers the morphogenetic type C as the plesiomorphic state for Leiuperinae (Fig 15F), but with an ambiguity at the node that relates it to Leptodactylinae. *Leptodactylus* embryos exhibit two different configurations: species of the *L*. *fuscus* group lack adhesive glands, whereas those of the *L*. *latrans* and *L*. *melanonotus* groups have type D glands defined as a medial heart-shaped structure [37]. Data on the *L*. *pentadactylus* group, and *Adenomera*, *Lithodytes*, *Hydrolaetare* and the Paratelmatobiinae are not available.

Type C glands are distinguished by an early division into two individual conical structures, and are known in several neobatrachian clades; e.g., some hylids [25], phyllomedusids [63], basal bufonids [52], batrachylids, and odontophrynids [60, 64]. We found remarkable variations in shape, size, and arrangement in the leiuperines (Figs 6 and 7; S1 Table). Glands of *Physalaemus* and *Pseudopaludicola* are usually conical, tall, and the diameter at the base is less than 3% of the body length, except for *Ph*. *albifrons* and *Ph*. *fernandezae* whose glands are slightly larger (3.3 and 3.7% respectively; Fig 6A–6D; S1 Table). At early stages glands are located lateroventral to the oral disc, later they become lateral, and regress almost at the level of the oral disc commissures. Conversely, in *Pleurodema* embryos the adhesive glands are rounded and low, with the largest diameters observed in our samples (about 5% of body length; Fig 6E and 6F). In early embryos they are ventral and very close to each other, and regress without changing their position relative to the oral disc. Gland regression in this genus occurs later than in the former two, i.e., when the oral disc and the spiracle are already developed and the hind limbs reach GS27 (Fig 14). Adhesive glands of *Pl*. *thaul* (Fig 7) are unusual in that the initial morphogenetic field has a V-shape configuration similar to that of the groove of other gland types (e.g., [37, 52]), and only acquire the typical arrangement for *Pleurodema* at later stages. This autapomorphy is certainly helpful for embryo recognition.

#### Hatching gland

The hatching gland is placed in the cephalic region and along a long dorsal line in most leiuperine species (Figs 8 and 9). The cephalic portion has a T-shaped configuration, progressing into two circular and lateral regions with dense, secretory cells with long microvilli, similarly to what has been described for *Leptodactylus* [37]. The hatching gland is conspicuously visible in embryos of *Pleurodema*, *Pseudopaludicola*, and in the pigmented embryos of *Physalaemus* (*Ph*. *riograndensis* and *Ph*. *cicada*). Conversely, in the remaining species of *Physalaemus* it is only visible with scanning electron microscopy. Although our sampling of this structure was not exhaustive (some stages were not imaged), we recorded a remarkable diversity in gland arrangements, dorsal extensions, shapes of secretory cells, and density and length of microvilli (Fig 8). In *Pseudopaludicola*, fully developed glands are represented by secretory cells with very short microvilli, disposed in a rostral transverse patch without a dorsal extension (Fig 8A and 8B). In *Physalaemus*, the hatching gland is arranged in a discontinuous patch of secretory cells with small microvilli (Fig 8C–8E). Rounded areas are lateral in the cephalic region and show a high density of secretory cells, and the dorsal line reaches midbody at advanced stages. In *Ph*. *fernandezae*, secretory cells are very small and mostly scattered, and only present close to the nares (Fig 9E). In *Pleurodema*, secretory cells are arranged in a large cephalic patch and in a dorsal line of variable length; some interspecific variations also involve the density of cells and the length of microvilli (Fig 8F and 8G). *Pleurodema borellii* presents the longest microvilli among leiuperines (Fig 8F), and in *Pl*. *bibroni* the dorsal line only reaches the anterior portion of the cephalic region. In the *Pl*. *thaul* clade the cells are sparsely distributed and not clustered in large groups. They have minute microvilli, and the dorsal line does not extend pass the gills level (Fig 9).

Hatching glands reach full development shortly before hatching, when cells become denser and microvilli length generally increases. As indicated by pigmentation, gland regression occurs in a postero-anterior direction, thus the frontal area is the last region to lose hatching cells. *Pleurodema cordobae* and species of the *Pl*. *nebulosum* group exhibit the latest regression of leiuperines, approximately at GS26–27.

#### Oral disc

Descriptions of the larval oral discs are available for the majority of leiuperine species, but data on their ontogenetic trajectories are scarce ([35]). The plesiomorphic and most generalized labial tooth formula includes two upper and three lower labial ridges. While the morphology of the upper lip is highly conserved, the number of ridges of the lower lip varies, and a reduced formula with two lower ridges evolved in the *Physalaemus biligonigerus* group, *Pleurodema tucumanum* and the *Pl*. *nebulosum* clade, and in several species of *Pseudopaludicola* including *Ps*. *mystacalis* in which it is polymorphic (Fig 16A). Marginal papillae of the lower lip are also variable, and may appear complete or interrupted by ventral and/or ventrolateral gaps.

In a research that included 12 leiuperine species, Vera Candioti et al. [35] identified varied developmental pathways related to the diversity of oral configurations within the group. According to their results, *Physalaemus* and *Pseudopaludicola* are characterized by the occurrence (transient or maintained in larval stages) of ventrolateral gaps, whereas in *Pleurodema* marginal papillae develop only from commissures and thus ventrolateral gaps never define. Additionally, the five types of oral disc known for *Physalaemus* result from common ontogenetic trajectories ending at different states, or from trajectories that differ initially in the formation of a ventral gap (see also Fig 10). Labial ridges are formed in slightly different ways among genera (Fig 14). In *Physalaemus* and *Pseudopaludicola*, the first ridge to differentiate is row P1 as an indented margin, and then row P2 forms as a transverse division along the ridge. Conversely, in *Pleurodema* row P2 develops first, and soon afterwards row P1 forms in a proximal direction. This is followed later by tooth emergence, which occurs first in older ridges.

Among the species we analyzed, the oral discs of *Physalaemus* aff. *albonotatus* and *Ph*. *albifrons* develop identically to those of other species of the *Ph*. *cuvieri* group ([35]; Fig 10). Conversely, oral ontogeny of *Ph*. *carrizorum* and *Ph*. *cicada* differs from the known trajectories. In *Ph*. *carrizorum*, the larval oral configuration shows three lower labial ridges and continuous lower papillae as described in other species of the *Ph*. *gracilis* group. However, the oral ontogenetic trajectory is similar to that of the *Ph*. *henselii* group, since it involves the initial presence of a ventral gap that, along with ventrolateral gaps, later complete with papillae (Fig 10A). Additionally, the P3 ridge develops before the mental papillae are outlined. Event sequence analysis showed that the larval oral disc acquires its definitive configuration earlier than in other species of the genus (Fig 14). In turn, *Ph*. *cicada* exhibits two larval oral configurations that develop following two different trajectories. A configuration with a ventral gap was previously described [65], and in half of our samples we observe comparable transient morphologies (e.g., Fig 10B); thus in these embryos we interpret an ontogenetic trajectory similar to that of the *Ph*. *henselii* group. In the other half, specimens show a different trajectory that ends in complete marginal papillae; in this case from the configuration with row P3 and both ventral and ventrolateral gaps, mental region completes first (Fig 10C). A transient oral morphology including the P3 row and only ventrolateral gaps was not reported for *Physalaemus*, but interestingly, it corresponds to the larval configuration of the recently described *Pseudopaludicola jaredi* [66].

In *Pseudopaludicola*, the oral disc ontogeny of *Ps*. *mystacalis* differs from what was described for *Ps*. *falcipes* (i.e., a trajectory almost identical to that of species of the *Physalaemus cuvieri* group; [35]). In most specimens, only two lower tooth ridges form and ventrolateral gaps persist in larval stages (Fig 11A–11D) [19]. A single specimen showed labial teeth on a mental papilla/ridge defining an incipient P3 (Fig 11E), a polymorphism also reported in *Ps*. *falcipes*. Additionally, a significant number of specimens (30%) have a wide ventral gap resembling bufonid tadpoles (Fig 11F). This oral configuration has been previously described for *Ps*. *boliviana* and *Ps*. *pusilla* [67–68].

The oral disc ontogeny in *Pleurodema* species was synthesized by Vera Candioti et al. [35], and it is simpler than those of *Physalaemus* and *Pseudopaludicola*. Data on five additional species in our study allow to discuss further variations among main clades. Embryos of *Pl*. *nebulosum* develop only two lower tooth ridges in a sequence similar to that of its sister species *Pl*. *guayapae*. In all other *Pleurodema*, three lower labial ridges appear. In *Pl*. *cordobae*, as in *Pl*. *bibroni* of the same intrageneric group, row P3 is very short and coexists briefly with a short ventral gap (Fig 12). As already stated [35], the marginal papillae in the genus develop from commissures and progresses in a medial direction, being the mental region the latest to complete. Alignment of marginal papillae is also variable. In *Pl*. *nebulosum* and *Pl*. *guayapae*, papillae are disposed in a single row, whereas in most other species they first appear widely spaced, and small gaps between them fill progressively resulting in alternate papillae (Fig 13). Finally, marginal papillae in the *Pl*. *bibroni* clade have the longest development in the genus, with an early differentiation of the first papillae and delayed acquisition of the larval configuration.

The ancestral state reconstruction of ventral and ventrolateral gaps allows for an interpretation of oral disc evolution that slightly differ from previous discussions by Vera Candioti et al. [35] and Lourenco et al. [46]. First, our sampling of *Physalaemus* does not include species of the *Ph*. *signifer* clade; consequently what we recover as synapomorphies of the genus may be so at the level of *Ph*. *cuvieri* clade, as already suggested [46]. Second, intraspecific variations (e.g., in *Ph*. *cicada* and *Pseudopaludicola* species) turn the reconstruction ambiguous at corresponding nodes. Third, developmental trajectories of newly sampled species (e.g., *Ph*. *carrizorum*) reveal unknown apomorphic features. Having said this, in our study we found that from a plesiomorphic absence, transient ventrolateral gaps evolve in the ancestor of *Physalaemus* (Fig 16B). These gaps persist in *Ph*. *riograndensis* and (as already stated in [46]) as a synapomorphy of the *Ph*. *cuvieri* group. In *Pseudopaludicola*, the sampling is still incomplete and includes polymorphic species, which makes the scenario uncertain. Nevertheless, given that ventrolateral gaps are never present in *Ps*. *boliviana*, *Ps*. *pusilla*, and some specimens of *Ps*. *mystacalis*, this feature does not characterize the genus as previously suggested [35]. A transient ventral gap occurs in most species of Leptodactylidae because of the way marginal papillae develop (only from commissures; Fig 16C). A larval ventral gap appears in the ancestor of *Physalaemus*, but it is soon lost at the most derived clade joining the *Ph*. *gracilis* and *Ph*. *biligonigerus* groups. A further change within that clade occurs in *Ph*. *carrizorum* embryos that show a reversal to the plesiomorphic, transient state. Knowledge of the ontogenetic trajectories for species belonging to the *Ph*. *signifer* clade, and the sister genera *Edalorhina* and *Engystomops* are crucial to resolve the ambiguities in the internal nodes of Leiuperinae, and establish the level at which these characters are apomorphic.

#### Hatching

We found substantial interspecific variation in the hatching time of leiuperine embryos (Fig 14; S1 Table). *Pseudopaludicola* experiences the earliest hatch, which occurs when gill arches are still differentiating (GS17–18). In *Pleurodema* and *Physalaemus*, most embryos hatch with long gills and operculum at their bases (GS21–22), but *Pl*. *nebulosum*, *Pl*. *diplolister*, species of the *Pl*. *bibroni* clade, *Ph*. *fernandezae*, *Ph*. *riograndensis* and *Ph*. *carrizorum* hatch relatively early. We observed some intraspecific variation as well in *Pl*. *borellii* and five species of *Physalaemus*. Such variation is likely underestimated since in nine of the species studied we only examined development from a single clutch.

### Evolution of early ontogenetic trajectories in Leiuperinae

Aside from structural diversity, we found phylogenetical informative shifts in the relative timing of developmental events (S2 Table). The optimization of developmental sequences on the hypothesized reference phylogeny resulted in a single most parsimonious reconstruction with 246 sequence heterochronies (Fig 17). Events with the highest number of shifts are the acquisition of the larval configuration of marginal papillae, the differentiation of labial ridge P3, the equaling of tail and body length, and the differentiation of the operculum at the gill base. In contrast, the events involving gill regression (concealing of the right and left gill. and spiracle development) are among the least variable. They define the Gosner Stages 24 and 25, and are traditionally considered the beginning of larval life. Their stability in relation to other external morphological features renders them useful landmarks for comparative analyses in leiuperines. Also, the negligible intraspecific variation in these events shows that phenotypic plasticity is not critical in these species (as it is in others, e.g., *Agalychnis callidryas*; [69]), at least in our seminatural breeding conditions.

Several heterochronic shifts in developmental sequences are recovered as synapomorphies (now on heterochronic synapomorphies) in the most recent common ancestor of Leiuperinae. They include the acceleration of tail growth, two events during gill development, and oral disc definition (Fig 17). The initial lengthening of the tail shows several changes across the tree, and it seems to be related to embryo/tadpole size. In general, a delayed equaling of tail and body lengths produce small embryos, and these in turn grow into small tadpoles (S1 Table). Observations in bufonid species show that embryo, tadpole, and adult sizes are not always directly correlated [52]. Regarding gill development, the medial fusion of the operculum often demarcates the onset of gill regression. Early full gill development followed closely by operculum medial fusion may be interpreted as a developmental change that shortens the functional period for the external gills. Heterochronic changes in gill development occur in *Leptodactylus*, specifically in the differentiation of the second pair. Departing from a delayed development synapomorphic for the genus, an acceleration occurs in the ancestral node of the *L*. *fuscus* group. This is consistent with what is known about gill morphology in *Leptodactylus*, in which the great development of the first pair at expenses of the other pairs in the *L*. *latrans* group contrasts with the poor but evenly developed pairs in the *L*. *fuscus* group [37]. The last events during oral disc ontogeny, i.e., the differentiation of row P3 and marginal papillae, are delayed in Leiuperinae. Nevertheless, this does not affect the acquisition of the definitive larval oral disc, which occurs earlier than in *Leptodactylus*. These changes are followed in more derived taxa by further developmental acceleration of other mouthparts and the coiling of the digestive tract, possibly related to an earlier onset of active feeding in some groups.

Within the Leiuperinae, the genus *Pseudopaludicola* is defined by a synapomorphic acceleration in tooth row P1 differentiation combined with a delayed P3 development (Fig 17). These two features are recovered independently in the ancestral ontogeny of *Physalaemus* (see below). A delayed tail growth is also characteristic of *Pseudopaludicola* species, intensified in *Ps*. *falcipes* that has the smallest embryos and tadpoles in our sample (S1 Table). *Pseudopaludicola* species also share a particular arrangement of the hatching gland, small adhesive glands, and small, scarcely branched gills that develop and regress early. This latter is intriguing, given the oviposition as loose eggs at the bottom of warm, still water bodies. A different mechanism for gas exchange is expected, and the small, slender body would be helpful for oxygen diffusion through the skin (e.g., [70]). Comparison of autapomorphic changes between *Pseudopaludicola* species should be discussed with a more complete representation of the genus diversity. In particular, variations in oral developmental trajectories are surely underestimated given the wide range of known larval oral configurations (e.g., see references in [35]).

Initial diversification in the subfamily also includes reproductive aspects. A foam nest built during the amplexus evolved in the sister group of *Pseudopaludicola*, the large clade joining *Pleurodema*, *Edalorhina*, *Engystomops*, and *Physalaemus* [45]. The behavior of building foam nests is a common a strategy among species that inhabit environments with unpredictable precipitation distribution and erratic availability of temporary ponds. It is therefore advantageous for those species to adapt to a larger diversity of habitats, to disperse and diversify [71]. Foam plays a main role in the protection of the egg mass from desiccation, temperature fluctuations, sun damage, predators, and in the oxygen diffusion and storage (revised in [6]). The relationships with embryo morphological and developmental characteristics are however hard to trace, and early studies have suggested that ecomorphological correlations may not relate so much to life in foam nests but with the length of time embryos spend there before they enter to the water [72].

In foam-nesting species of the clade *Pleurodema* + *Physalaemus*, tailbud embryos are widely diverse in size, shape, yolk provision, and pigmentation (Fig 1; see also [23–24, 32, 61]). This variation has in principle a strong phylogenetic structure, with most *Physalaemus* tailbud embryos being small, white, and kyphotic, in contrast to *Pleurodema* embryos that are large, highly pigmented, and straight. However, some patterns appear to occur in relation to specific variations in oviposition. Dark pigmentation may play a role in camouflage, protection against solar UV radiation, and intake of solar heat required to accelerate embryo development [5]. Consequently, eggs that develop in concealed, shady places (e.g., clutches in leaves, under rocks, within the parental body) are usually unpigmented, whereas eggs laid in open and aquatic environments are often highly pigmented [34, 56]. Likewise, large-sized and yolky embryos typically occur in groups with endotrophic development [73], but also in exotrophic forms that go through long intracapsular periods, such as some phyllomedusids, centrolenids, and foam-nesting rhacophorids [74–76]. Large, white, heavily yolked embryos of the *Leptodactylus fuscus* group hatch early (both in time and developmental stage; [26, 37]) but remain within the foam nests depending solely upon yolk store until they reach the water [77]. Conversely, foam embryos of *Pleurodema* and *Physalaemus* hatch early and also leave the foam nest soon after hatching (e.g., [78]). The diversity of pigmentation, size, and shape of embryos in this clade can be also related to local environmental conditions (e.g., temperature, permanence) of the water bodies where the free-living embryos develop. The large clade *Pleurodema* + *Physalaemus* is also characterized by an accelerated growth of the hind limbs (Fig 17). Proportionately large and precocious hind limbs evolved also in foam-nest *Leptodactylus*, especially in the *L*. *fuscus* group with terrestrial foam nests [6]. Several taxa distant to Leptodactylidae also show precocious hind limb differentiation, in many cases related to terrestrial or endotrophic development (e.g., *Batrachyla*, *Dendrophryniscus*, *Eupsophus*; [52, 64, 79]).

The genus *Pleurodema* is diagnosed by an early differentiation of tooth row P2, early coiling of the digestive tract, and delayed growth of the tail, gills, and spiracle (Fig 17). Interestingly, the four latter events are reversed within the derived lineage joining the *Pl*. *bibroni* and *Pl*. *thaul* clades. This lineage is characterized by a loss of the foam nest, and darkly pigmented eggs are laid instead in jelly masses or strings [45]. The clade includes species that inhabit high latitudes (Patagonian clade *Pl*. *thaul*; [15–16]) or breed at lower temperatures than cogeneric taxa (*Pl*. *bibroni* clade; [12–13]). Several features of these species are apparently related to development in fresh, highly oxygenated environments. First, eggs laid in cold water tend to have large gelatinous capsules, which render a large total egg to ovum ratio when compared with species that develop in warmer environments (e.g., ratios 4.1 vs. 1.6 in ranoid *Rana japonica* and *Fejervarya cancrivora*, respectively; [5]). In species we studied, the proportional size of jelly capsules of *Pl*. *bufoninum* and *Pl*. *thaul* is appreciably larger than that of *Pl*. *guayapae* eggs (ratios 1.9–3.4 vs. 1.5–1.7; see also [16, 80]). A relationship between the jelly thickness and light refraction, which would produce a lens effect for heat conduction, has been suggested [5] and could be further explored in this group. In second place, large embryos and early tail lengthening in these species are consistent with general patterns in larval ectotherms, where low temperatures favor size increase without the tissue differentiation required by stage progression [81].

Low water temperature has a well-established relationship with oxygen solubility, and this in turn often shows a morphological correlate in breathing structures. Correlations between external gill size and branching and oxygen availability in water bodies have been explored in amphibians (e.g., [82–83]), and experimentally shown within some species (e.g., [84]). Likewise, body ciliation is considered to have an important respiratory role both prior and after hatching [28]. In this context, we interpret the short, scarcely branched gills with brief development, and the overall sparse and ephemeral ciliation of embryos of cold water breeding *Pleurodema* species as associated to high tension of oxygen in the water. Finally, a delayed gut coiling is linked with a general initial larger amount of yolk in these and other distant species [64, 73]. While in endotrophic species a delayed development of the digestive tract is related to the sole dependence on yolk for embryo nutrition, in exotrophic species it likely derives in a later onset of active feeding. In this regard, activity patterns, with feeding rates among them, are known to have a direct relation to temperature variations in larval anurans (e.g., [85]). Most of these aforementioned structural and heterochronic shifts evolve convergently in the *Physalaemus henselii* group, whose species breed in similar environmental conditions but within foam nests [21–22]. This reinforces the hypothesis that morphological and developmental evolution in leiuperines included a distinct ecomorphological component related to local abiotic factors.

On the other extreme, the *Pleurodema nebulosum* clade includes species that inhabit arid environments and reproduce in temporary, brackish ponds at high temperatures (e.g., [86]). These embryos are among the smallest and less yolked in the genus, and are profusely ciliated, with large ciliated cells that regress later after the hind limb bud emergence. They also have comparatively well-developed, highly branched gills, and a reduced labial tooth row formula with only two lower labial ridges. In reconstructed ancestral ontogenies, the clade is diagnosed by accelerated hind limb emergence and early differentiation of mouthparts (Fig 17). Some similar features (small size, large gills, early hind limb and mouthparts differentiation) evolved independently in *Pl*. *diplolister* and *Physalaemus cicada* that breed in arid environments of the Brazilian Caatinga [20, 87]. Reproduction in xeric, high temperature conditions imposes risks of desiccation and insolation, and thus it is often associated with short development to metamorphosis (but see also [88–89)]. Some morphological and heterochronic features of embryos in our study can be interpreted as pre-requisites of accelerated development, or as part of specific mechanisms to deal with gas and ionic exchange in highly temporary environments.

Embryos of *Pleurodema guayapae* and *Pl*. *diplolister* have very short developments, with early hatching about one day after oviposition [9–10] and metamorphosis completed in 2–3 weeks in *Pl*. *diplolister* [90] and in an exceptionally brief lapse of 9–10 days in *Pl*. *guayapae* (J. Lescano pers. comm.). A comparable short development is described for other desert species such as ceratophryids, some scaphiopodids, and pyxicephalids [87, 91–92]. Along with an overall condensed premetamorphic life, some individual features undergo heterochronic shifts to precocious differentiation. As in *Pl*. *nebulosum* clade, hind limbs emerge early in *Lepidobatrachus laevis* and other ceratophryid embryos ([93]; J. Grosso et al. unpubl. data). The effect of thyroid hormones on several events that occur during metamorphosis, e.g., limb development, has been deeply studied (e.g., recent reviews in [94–96]), and a precocious activation of thyroid and interrenal axes under pond desiccation was experimentally shown in *Spea hammondii* [97]. In this context, a faster development of limbs would be consistent with an early appearance of some adult-like features (e.g., a posterior jaw suspension) reported in other short-developing frogs (e.g., [90, 98]). The evolution of keratinized mouthparts, more flexible and easily-discarded than hard tissues like dentin and enamel, has been interpreted as a suitable strategy for transient stages [99]. An early differentiation along with a fast regression could be particularly critical in unstable environments. This could explain the heterochronic acceleration of mouthparts development and the reduction of the labial tooth row formula in the *Pl*. *nebulosum* clade. This pattern however cannot be verified in other *Pleurodema* species (e.g., although Chacoan *Pl*. *tucumanum* do show labial tooth row formula 2/2, *Pl*. *diplolister* maintains the plesiomorphic state 2/3; [89, 100]) nor in the unrelated, desert tadpoles of *Scaphiopus* [101].

Breathing structures (both body ciliation and gill features) in these species appear to show a pattern inverse to that commented for embryos developing at cold temperatures. In addition, a role in ionic exchange has been discussed for larval amphibian gills (compiled in [102]). Based on ultrastructural observations, Uchiyama and Yoshizawa [103] rule out the external gills from a salinity tolerance mediated by mitochondria-rich cells, and postulate that higher concentrations of Na^+^ and Cl^-^ in body fluids would explain the resistance of posthatching embryos of *Fejervarya cancrivora* to up to 40% seawater. Species of the *Pleurodema nebulosum* clade breed in saline ponds, and structural and physiological mechanisms of tolerance and acclimation to salinity would be certainly worth to explore in this group.

The ancestral trajectory of *Physalaemus* included four synapomorphic transformations in mouthparts development (Fig 17). An early differentiation of row P1 and delayed definition of row P3 occur convergently with *Pseudopaludicola*, and this is consistent with a sequential appearance of the three lower ridges in both taxa [35]. Additionally, in *Physalaemus* row A2 and marginal papillae acquire prematurely their larval configuration. This feature is interesting, as oral disc morphology is complex in this genus (in comparison to *Pleurodema*), with a variety of arrangements of gaps and papillae. The accelerated differentiation of these structures, along with the overall low intraspecific variations in most species, seems to indicate an underlying highly adjusted developmental mechanism. Adhesive glands show some heterochronic changes only in this genus. An early division occurs in *Ph*. *henselii*, and independent accelerations of gland regression take place in the ancestral trajectories of the clades *Ph*. *cuvieri* + *Ph*. *albifrons* and *Ph*. *cicada* + *Ph*. *biligonigerus*. This overall accelerated development of adhesive glands could be associated to their relative smaller sizes compared to those of *Pleurodema*. The *Ph*. *cuvieri* group is characterized by a secondary acceleration in the definitive configuration of the marginal papillae. The paedomorphic oral morphology of these species (i.e., with persisting ventrolateral gaps) is similar to transient states of the oral trajectories in the sister clade *Ph*. *henselii*, thus its early definition could be interpreted as a consequence of truncated development. Likewise, a further accelerated formation of marginal papillae, followed by an early definition of the oral disc, is recovered as a synapomorphy of the *Ph*. *biligonigerus* group, which contains the species with the simplest oral configurations in the genus. Unlike the *Ph*. *cuvieri* group, the paedomorphic oral disc of *Ph*. *riograndensis* does not show heterochronic shifts regarding the ancestral trajectory of the sister species with complete ventrolateral gaps. A more refined interpretation of the role of sequence heterochronies in the evolution of oral disc diversity in *Physalaemus* would require a more detailed coding of events during marginal papillae development, and the inclusion of representatives of the *Ph*. *signifer* clade. Finally, some events related to gill development (including the differentiation of the third pair) are decelerated in the clade *Ph*. *gracilis + Ph*. *santafecinus*, and next in the ancestral node of the *Ph*. *biligonigerus* group. This is apparently unrelated to gill overall structure.

## Conclusions

The Leiuperinae have a vast diversity in morphological and developmental aspects of embryonic features. This diversity helps to reveal the evolution of these frogs at both the generic and intrageneric levels. Intraspecific variation is overall low, except within the oral ontogeny of *Pseudopaludicola* spp. and *Physalaemus cicada*. In general, embryonic features of *Physalaemus* and *Engystomops* are derived in the subfamily, and some are unique (kyphosis and lack of pigmentation) but others, especially those of the oral region, occur convergently in embryos of *Pseudopaludicola* (ventrolateral gaps, early row P1, delayed row P3, conical adhesive glands). A few morphological features are relatively conserved along the tree or within groups. For instance, the adhesive glands, although variable in the sister clade Leptodactylinae, are universally present with the type-C configuration in the Leiuperinae; slight size and developmental timing variations occur between *Physalaemus* and *Pleurodema*. Likewise, the presence of row P3 is plesiomorphic for the subfamily, only changing in derived clades of *Physalaemus* and *Pleurodema*, and in several species of *Pseudopaludicola* not included in this work. The number of gill pairs is conserved in *Pleurodema* and the *Ph*. *cuvieri* clade, but varies in other groups, making it impossible to elucidate ancestral states along the whole tree. Ciliation and hatching glands were not examined as intensively as other features (some developmental stages and body areas are missing) to allow an accurate quantification. Whereas ciliation shows some patterns that outline ecomorphological relationships, we found no clear relationship between variations in hatching gland structure and hatching time. Covariation between morphology, development, and the ecology of embryos is observed for at least some of these species, but further field observations and experimental assessments are required. We identify convergent patterns for embryos developing in cold, oxygenated environments, which involve a large body size, poorly developed transient respiratory structures, large yolk provision, and a delayed development of the digestive tract. Conversely, embryos that develop in warm, brackish water bodies of xeric environments show more complex and persistent gills and body ciliation, and faster hind limb development. Our survey highlights that morphology and early development of anurans can be a valuable source of information for phylogenetic studies, and provide fundamental bases to explore and discuss how evolutionary changes can also be shaped by environmental conditions.

## Supporting information

S1 Appendix Material examined. (DOCX)

S2 Appendix Script used for execute the R-package Pgi2. (PDF)

S3 Appendix Matrix of ranks for sequence heterochrony analysis. (PDF)

S4 Appendix Matrix of discrete and continuous characters. (PDF)

S5 Appendix Matrix of development sequence for the R-package Pgi2. (PDF)

S1 Fig Embryonic measurements and morphological features recorded. (PDF)

S1 Table Summary of morphological characters in leiuperine embryos. (PDF)

S2 Table Sequences of developmental events in species of Leiuperinae. (PDF)
