# Supplementary material for: Early ontogeny and sequence heterochronies in Leiuperinae frogs (Anura: Leptodactylidae)
Source: PLoS One. 2019 Jun 27;14(6):e0218733. doi: 10.1371/journal.pone.0218733 (PMC6597095; doi:10.1371/journal.pone.0218733)
Supplement: S1 Appendix — (DOCX) [file pone.0218733.s001.docx]

**S1 Appendix. Material examined.** Voucher numbers and localities uses for this study. Each clutch has a voucher number, the species were collected with the approval of the national and regional authorities: Argentina, Dirección de Flora y Fauna (81/2015 and 01/2017 –DS y FS), Secretaría de Medioambiente (N° 41-AP-2014), Dirección Provincial de Áreas Naturales Protegidas (14/16), Secretaria de Ambiente from Ministerio de Ambiente y Producción Sustentable (EXPTE 0090227-13596/2014-0), Dirección Flora, Fauna Silvestre y Suelos (EXPTE 1865-330-P-2,015), Ministerio de Ecología y Recursos Naturales Renovables (MEyRNR, 007/2009, 048/2013, 072/2014, 061/2015, 073/2016, and 035/2017), Programa de Recursos Naturales y Medio Ambiente (PRNyMA, 01/2016); Brazil, SISBIO (N.º 60078-3 Código de autenticação: 0600780320181101); Uruguay, División Fauna, Ministerio de Ganadería, Agricultura y Pesca (Res. Nº 199/13 and 137/16).

*Physalaemus cicada:* BRAZIL: Bahia: Brotas de Macaúbas (LGE 21630: N= 80, GS 16–26).

***Ph. biligonigerus* group**

*Ph. biligonigerus:* ARGENTINA: Chaco: General Güemes: 27.5 kilometers southeast to Misión Nueva Pompeya (LGE 11847: N= 100, GS 17–26; LGE11849: N= 20, GS 25–26; LGE11850: N= 40, GS 16–22); Córdoba: Colón: Pozo Azul (LGE 10456 and FML30440: N= 10, GS 16–17); Río Seco: Ruta Provincial Nº 9, near to interprovincial boundary Córdoba-Santiago del Estero (LGE 5827: N= 50, GS 17–25); Misiones: Apóstoles: Ruta Provincial Nº 1, 5.6 kilometers norwestern to Azara (LGE 6099: N= 90, GS 16–26; LGE6100: N= 10, GS 21–23; LGE6101: N= 120, GS 17–18; LGE6102: N= 30, GS 22–26; LGE6103: N= 30, GS 22–26); San Javier: Ruta Provincial Nº 2, 1 kilometers southern to Arroyo Toribio (LGE 7327: N= 120, GS 16–26); Tucumán: Tafí Viejo, El Cadillal (LGE 146 and FML30430: N= 90, GS 19–26); Santiago del Estero: Juan Felipe Varela: Suncho Corral (LGE 7974: N= 60, GS 19–23); Loreto: Totora Pampa, Ruta Nacional Nº 9 and Ruta Provincial Nº 1 (LGE 10453 and FML30438: N= 60, GS 16–26; LGE10454 and FML30439: N= 60, GS 17–26).

*Ph. riograndensis*: ARGENTINA: Misiones: Candelaria: Ñu Pyahú (LGE 3407 and FML30431: N= 30, GS 21–26; LGE3487: N= 90, GS 17–26; LGE3488: N= 80, GS 17–26); near to Profundidad (LGE 7200: N= 100, GS 16–26; LGE7201: N= 80, GS 21–26; LGE7002: N= 80, GS 16–26); Capital: Garupá, Barrio Santa Helena (LGE 14012: N= 50, GS 20–24). Uruguay: Treinta y Tres: Ruta entre Sierra del Tigre y Treinta y Tres (LGE 1796: N= 50, GS 20–26).

*Ph. santafecinus*: ARGENTINA: Corrientes: Capital: Laguna Brava, Barrio Dos Lunas, Don Luís (LGE 93 and FML30428: N= 40, GS 21–24); Ituzaingó: Ituzaingó (LGE 4951: N= 90, GS 17–25; LGE4952: N= 100, GS 17–26; LGE4953: N= 90, GS 17–25; LGE4954: N= 100, GS 17–26; LGE4955: N= 80, GS 17–26; LGE4956: N= 100, GS 17–26, LGE 7005: N= 100, GS 16–26; LGE7006: N= 100, GS 16–26); Santa Fe: La Capital: San José del Rincón, Villa California, Calle del Sol (LGE 216293 and FML30447: N= 30, GS 17–23).

***Ph. gracilis* group**

*Ph. gracilis:* URUGUAY: Montevideo: Montevideo, Parque Vaz Ferreira (LGE 1797*:* N= 40, GS 21–26).

*Ph. carrizorum*: ARGENTINA: Misiones: San Pedro: Parque Provincial El Piñalito (LGE 20430: N= 50, GS 17–26).

***Ph. cuvieri* group**

*Ph. albonotatus*: ARGENTINA: Chaco: General Güemes: El Sauzalito (LGE 10457 and FML30441*:* N= 20, GS 18–22); 27.5 kilometers southeastern to Misión Nueva Pompeya (LGE 11846: N= 50, GS 16–23; LGE11848: N= 120, GS 16–26); Jujuy: El Carmen: 2 kilometers northern to Pampa Blanca (LGE 10462 and FML30444; LGE10463 and FML30445*:* N= 20, GS 16–17; LGE10465 and FML30446: N= 10, GS 17–18).

*Ph.* aff. a*lbonotatus*: ARGENTINA: Corrientes: Ituzaingó: Ituzaingó (LGE 7004 and FML30433: N= 30, GS 17–24).

*Ph. cuvieri:* ARGENTINA: Misiones: Cainguás: Aristóbulo del Valle, Balneario del Arroyo Cuña Pirú (LGE 143 and FML30429: N= 30, GS 17–26); Capital: Garupá, Barrio Santa Helena (LGE 14005: N= 70, GS 16–26; LGE 14007: N= 60, GS 17–26; LGE 14009: N= 100, GS 17–26; LGE14014: N= 100, GS 17–26); Eldorado: Santiago de Liniers, Reserva Natural La Emilia, Establecimiento Don Guillermo (LGE 7074 and FML30434: N= 100, GS 17–26; LGE7350: N= 10, GS 16–17); General Manuel Belgrano: Lote J19, 10 kilometers northern to Dos Hermanas (LGE 4833: N= 40, GS 16–22). BRAZIL: Bahia: Barreiras (LGE 21631: N= 60, GS 17–25).

*Ph. albifrons*: BRAZIL: Bahia: Brotas de Macaúbas (LGE 21632: N= 70, GS 17–26).

***Ph. henselii* group**

*Ph. fernandezae:*ARGENTINA: Buenos Aires: Ensenada: Punta Lara (LGE 1794: N= 40, GS 19–26).

*Ph. henselii*: URUGUAY: Rocha: Rocha, Ruta N° 10, kilometer 255 (LGE 1795: N= 30, GS 20–26; LGE14680: N= 15, GS 23–26); Treinta y Tres: Sierra del Tigre (LGE 14681: N= 20, GS 20–26).

***Pleurodema bibroni* clade**

*Pl. bibroni*: URUGUAY: Rocha: Rocha, Ruta N° 10, kilometer 255 (LGE 1798: N= 30, GS 18–26).

*Pl. cordobae*: ARGENTINA: Córdoba: Calamuchita: estancia Los Tabaquillos: (LGE 9260: N= 50, GS 16–26; LGE9277: N= 50, GS 19–24; LGE10470: N= 10, GS 21–23).

***Pl. brachyops* clade**

*Pl. borellii*: ARGENTINA: Jujuy: Doctor Manuel Belgrano: Tiraxi (LGE 10461 and FML30443: N= 20, GS 17–21); Tucumán: Capital: San Miguel de Tucumán, Fundación Miguel Lillo (LGE 144: N= 20, GS 23–26; LGE6226 and FML30432: N= 120, GS 17–26); Yerba Buena (FML 29854: N= 10, GS 23–26); Lules: Near to Lules, Ruta Provincial N° 341 (LGE 10458 and FML30442: N= 30, GS 18–22); San Javier, Calle 4 (LGE 15573: N= 25, GS 23–26).

*Pl. diplolister:* BRAZIL: Bahia: Brotas de Macaúbas (LGE 21633: N= 90, GS 17–26).

***Pl. thaul* clade**

*Pl. bufoninum*: ARGENTINA: Río Negro: 25 de Mayo, Ruta Provincial Nº 8 water shed at 19km NE (CPN-A 2975: N= 80, GS 17–26).

*Pl. thaul*: ARGENTINA: Rio Negro: Bariloche: Bariloche, Llao Llao (LGE 14673: N= 20, GS 17–22; LGE 14674: N= 20, GS 17–22; LGE 14675: N= 100, GS 16–26; LGE14676: N= 100, GS 16–26).

***Pl. nebulosum* clade**

*Pl. guayapae*: ARGENTINA: Santiago del Estero: Loreto: Totora Pampa, Ruta Nacional Nº 9 and Ruta Provincial Nº 1 (LGE 9763 and FML30435: N= 30, GS18–25; LGE10227: N= 20, GS 23–26; LGE10240 and FML30436: N= 40, GS 18–23; LGE10254: N= 50, GS 18–23; LGE10452: N= 10, GS 16–23; LGE 10455 and FML30437: N= 20, GS16–19).

*Pl. nebulosum:* ARGENTINA: Catamarca: Santa María: Ruta Nacional Nº 40 near to Pie de Médano (LGE 2368: N= 40, GS 23–26).

***Pseudopaludicola***

*Ps. falcipes*: ARGENTINA: Misiones: Candelaria: Candelaria, Arroyo Garupá and Ruta Provincial Nº 204 (LGE 20275: N= 50, GS 17–26); near to Profundidad (LGE 7203: N= 80, GS 17–25). URUGUAY: Artigas: Bella Unión (LGE 14691: N= 10, GS 17–19); Treinta y Tres: Treinta y Tres (LGE 1799: N= 50, GS 21–26).

*Ps. mystacalis:* ARGENTINA: Misiones: Capital, Arroyo Pindapoy Chico (LGE 6098: N= 60, GS 18–26).
