# Supplementary material for: Early ontogeny and sequence heterochronies in Leiuperinae frogs (Anura: Leptodactylidae)
Source: PLoS One. 2019 Jun 27;14(6):e0218733. doi: 10.1371/journal.pone.0218733 (PMC6597095; doi:10.1371/journal.pone.0218733)
Supplement: S2 Appendix — (PDF) [file pone.0218733.s002.pdf]

S2 Appendix. Script used for execute the R-package Pgi2. A loop for cycles, saving the best result found.

```
library(pgi2)

datos <- pgi.read.nexus(file="hetero.nex")
best <-
pgi(datos,nruns=c(1,1),inf.params=list(heuristic="pgi",cycles=1,replicates=1,ret.anc.seq=50,simultaneity=TRUE,edit.cost.func="parsimov"),con.params=list(con.type="simple",edit.cost.func="parsimov"),verbosity=1)
bLength <- best$tree.length

lenList <- NULL

for (i in 1:10) {
	res <-
pgi(datos,nruns=c(1,1),inf.params=list(heuristic="pgi",cycles=200,replicates=200,ret.anc.seq=50,simultaneity=TRUE,edit.cost.func="parsimov"),con.params=list(con.type="simple",edit.cost.func="parsimov"),verbosity=1)
	if (res$tree.length < bLength) {
		bLength <- res$tree.length
		best <- res
		save(best, file="best-r01.rda")
	}
	lenList <- cbind(lenList, res$tree.length)
	save(res, file=paste("r01-it", i))
}

# q(save="no")
```
