# Supplementary material for: Early ontogeny and sequence heterochronies in Leiuperinae frogs (Anura: Leptodactylidae)
Source: PLoS One. 2019 Jun 27;14(6):e0218733. doi: 10.1371/journal.pone.0218733 (PMC6597095; doi:10.1371/journal.pone.0218733)
Supplement: S3 Appendix — (PDF) [file pone.0218733.s003.pdf]

S3 Appendix. Matrix of ranks for sequence heterochrony analysis. Species: Oa *O. americanus*, Lc *L. chaquensis*, Pha *Ph.* aff. *albonotatus*, Phb *Ph. albifrons*, Phc *Ph. cicada*, Phf *Ph. fernandezae*, Phg *Ph. gracilis*, Phh *Ph. henselii*, Phi *Ph. biligonigerus*, Pho *Ph. albonotatus*, Phr *Ph. riograndensis*, Phs *Ph. santafecinus*, Phu *Ph. cuvieri*, Phz *Ph. carrizorum*, Plb *Pl. borellii*, Plc *Pl. cordobae*, Pld *Pl. diplolister*, Pli *Pl. bibroni*, Plg *Pl. guayapae*, Pln *Pl. nebulosum*, Plt *Pl. thaul*, Plu *Pl. bufoninum*, Psf *Ps. falcipes*, Psm *Ps. mystacalis* (specimens with oral lower papillation). Events: (N = 24): AG adhesive gland first visible, TB tailbud, 1G first gill pair bud, TL=BL tail length / body length = 1, 2G second gill pair bud, 1GB first gill pair branched, 2GB second gill pair branched, A1 labial tooth ridge A1, P2 labial tooth ridge P2, OB operculum at gill base, FP first marginal papillae, P1 labial tooth ridge P1, A2 labial tooth ridge A2, GFD gills at full development, OM operculum medially fused, IC first coil in digestive tract, RGC right gill covered by operculum, MP marginal papillae complete, LGC left gill covered by operculum, ES spiracle developed, HLB hind limb buds, AGA adhesive glands absent, LOD oral disc fully formed, HL26 hind limbs at GS26.

	Oa	*Lc*	*Pha*	*Phb*	*Phc*	*Phf*	*Phg*	*Phh*	*Phi*	*Pho*	*Phr*	*Phs*	*Phu*	*Phz*	*Plb*	*Plc*	*Pld*	*Pli*	*Plg*	*Pln*	*Plt*	*Plu*	*Psf*	*Psm*
AG	1.5	1.5	2	1.5	1.5	1.5	3	2.5	1.5	1.5	1.5	1.5	1.5	1.5	1.5	1.5	1.5	2	1.5	3	1.5	1.5	1.5	1.5
TB	1.5	1.5	1	1.5	1.5	1.5	3	2.5	1.5	1.5	1.5	1.5	1.5	1.5	1.5	1.5	1.5	1	1.5	3	1.5	1.5	1.5	1.5
1G	3.5	3	3	3	3	3	3	2.5	3	3	3	3	3	3	3	3	3	3.5	3	3	3	3	3	3
TL=BL	3.5	5.5	6.5	7.5	9	4.5	3	6	4.5	4.5	8	5.5	4.5	9	7	5	9	5	9	3	6.5	8	8	5
2G	5	10	4	4	4	4.5	3	2.5	4.5	4.5	4	4	4.5	4	4	4	4	3.5	4	3	4	5	4	4
1GB	6	4	5	5	5	6	8	6	6	6	5	5.5	6	5.5	5	6.5	5	10	5	8	5	4	5.5	8
2GB	7	12	6.5	6	6	11	8	6	7	8	6	7	7	7	6	9	6	10	6	8	10	6	10	11.5
A1	9	7.5	9	9.5	9	8	8	9	9.5	9.5	8	8.5	9.5	9	9	9	9	6.5	9	8	8.5	9.5	8	8
P2	9	7.5	9	12.5	11.5	10	11	12	11.5	9.5	12	11	11	11.5	9	9	9	6.5	9	8	8.5	9.5	12.5	11.5
OB	9	5.5	9	7.5	7	8	8	9	8	7	10	10	8	5.5	9	6.5	7	8	7	8	6.5	7	5.5	8
FP	11.5	14	13	12.5	13.5	14	13	14	11.5	13	14.5	12.5	14	11.5	12	11	13	10	11	11	14	14	12.5	15
P1	11.5	10	11	9.5	9	8	8	9	9.5	11	8	8.5	9.5	9	11	13.5	11.5	16.5	17.5	13.5	12.5	17	8	8
A2	14.5	10	13	12.5	11.5	12.5	13	12	16	13	11	12.5	12	13	13	13.5	11.5	12.5	17.5	16	11	13	15	17.5
GFD	14.5	17	13	12.5	16	12.5	13	12	13	13	14.5	16	13	14.5	15	15.5	15	12.5	12	13.5	12.5	11	11	8
OM	14.5	15.5	15	15	13.5	15	15	15	14	15	13	15	15	14.5	16	12	15	14	16	13.5	15	12	14	13
IC	14.5	13	16	16	15	16	16	17	15	16	16	14	16	16	14	15.5	15	16.5	13.5	13.5	18	15	17	14
RGC	17	19	18	17	17	17	18	16	17	17	17	17	18	17	17	17	18	15	19	18	16	16	16	16
MP	18	15.5	17	21.5	19.5	19.5	17	21	18	19	18	20.5	17	18.5	19	20	17	20	20.5	18	20	19	20	19.5
LGC	19	20.5	19	18	18	18	19	18	19	18	19	18	19	18.5	18	19	20	18	20.5	20	17	20	18	17.5
ES	20	22.5	20	20	19.5	19.5	20	19	20	20	20	19	21	20.5	21	21.5	21	19	22	21.5	19	21	19	19.5
HLB	21	18	21	23	21	23	21	20	23	21	21	20.5	20	22	22.5	18	19	21	13.5	18	21	18	21	21.5
AGA	22	24	24	19	22.5	21	23	24	21	22	24	22	22	23	24	24	23	24	24	24	23	24	23	23
LOD	23	22.5	22.5	21.5	24	22	23	22	22	23.5	22	23	23	20.5	20	23	22	22	23	23	22	23	22	21.5
HL26	24	20.5	22.5	24	22.5	24	23	23	24	23.5	23	24	24	24	22.5	21.5	24	23	15	21.5	24	22	24	24
