# Supplementary material for: Early ontogeny and sequence heterochronies in Leiuperinae frogs (Anura: Leptodactylidae)
Source: PLoS One. 2019 Jun 27;14(6):e0218733. doi: 10.1371/journal.pone.0218733 (PMC6597095; doi:10.1371/journal.pone.0218733)
Supplement: S4 Appendix — (PDF) [file pone.0218733.s004.pdf]

S4 Appendix. Matrix of discrete and continuous characters. Species: Oa *O. americanus*, Lb *L. bufonius*, Lc *L. chaquensis*, Lf *L. fuscus*, Lg *L. gracilis*, Ll *L. latinasus*, Lp *L. plaumanni*, Lr *L. latrans*, Ma *Melanophryniscus atroluteus*, Mk *M. krauczuki*, Ms *M. sanmartini*, Ec *Engystomops coloradorum*, Eg *E. guayaco*, Pha *Ph.* aff. *albonotatus*, Phb *Ph. albifrons*, Phc *Ph. cicada*, Phf *Ph. fernandezae*, Phg *Ph. gracilis*, Phh *Ph. henselii*, Phi *Ph. biligonigerus*, Pho *Ph. albonotatus*, Phr *Ph. riograndensis*, Phs *Ph. santafecinus*, Phu *Ph. cuvieri*, Phz *Ph. carrizorum*, Plb *Pl. borellii*, Plc *Pl. cordobae*, Pld *Pl. diplolister*, Pli *Pl. bibroni*, Plg *Pl. guayapae*, Pln *Pl. nebulosum*, Plr *Pl. brachyops*, Plt *Pl. thaul*, Plu *Pl. bufoninum*, Psf *Ps. falcipes*, Psm *Ps. mystacalis* (specimens with oral lower papillation). Characters (N = 9): TL length at taibud stage (in mm); TY yolk area at tailbud stage (in % of the body area), DA dorsal angle at tailbud stage (in degrees), TP pigmentation at tailbud stage (0 absent – 1 present), GP gill pairs (0 2 – 1 3), AG adhesive glands (0 absent – 1 type C – 2 type D), P3 row P3 (0 absent – 1 present) , VL ventrolateral gaps (0 absent – 1 transient – 2 definitive), VG ventral gap (0 absent – 1 transient – 2 definitive).

	Pha	*Phb*	*Phc*	*Phf*	*Phg*	*Phh*	*Phi*	*Pho*	*Phr*	*Phs*	*Phu*	*Phz*	*Plb*	*Plc*	*Pld*	*Pli*	*Plg*	*Pln*	*Plt*	*Plu*	*Psf*	*Psm*
TL	1.50	1.40	?	?	?	?	1.60	1.50	1.40	1.40	1.50	1.90	2.00	2.70	1.70	?	1.60	?	1.60	2.40	2.30	1.10
TY	0.42	0.34	?	?	?	?	0.40	0.38	0.48	0.47	0.41	0.47	0.41	?	0.41	?	0.39	?	?	0.46	0.47	0.30
DA	0.66	0.46	?	?	?	?	0.75	0.62	0.64	0.71	0.63	0.80	0.95	1.60	1.39	?	1.20	?	?	0.96	1.04	1.43
TP	0	0	0	0	0	0	0	0	1	0	0	0	1	1	1	1	1	?	0	1	1	1
GP	3	3	3	2	3	2	3	3	3	3	3	3	3	3	3	3	3	3	3	3	3	2
AG	1	1	1	1	1	1	1	1	1	1	1	1	1	1	1	1	1	1	1	1	1	1
P3	1	1	1	1	1	1	0	1	0	0	1	1	1	1	1	1	0	0	1	1	1	1
VL	2	2	1	1	1	1	1	2	2	1	2	1	0	0	0	0	0	0	0	0	0	2
VG	2	2	[12]	2	0	2	0	2	0	0	2	1	1	1	1	1	1	1	1	1	1	[02]

	Oa	*Lb*	*Lc*	*Lf*	*Lg*	*Ll*	*Lp*	*Lr*	*Ma*	*Mk*	*Ms*	*Ec*	*Eg*
TL	1.36	2.50	2.15	2.00	2.90	1.90	2.50	2.00	2.38	1.90	1.76	1.70	1.50
TY	0.30	0.59	0.39	0.59	0.63	0.50	0.57	0.44	0.36	0.25	0.48	0.42	0.33
DA	0.83	1.12	1.45	1.12	1.16	1.25	0.96	1.00	1.07	0.63	0.58	0.64	0.57
TP	1	0	1	0	0	0	0	1	1	1	1	0	0
GP	2	3	3	3	3	3	3	3	?	3	?	2	?
AG	1	0	2	0	0	0	0	2	1	1	1	1	1
P3	1	1	1	1	1	1	1	1	1	1	1	1	1
VL	0	0	0	0	0	0	0	0	0	0	0	[01]	[01]
VG	1	1	1	1	1	1	1	1	1	1	1	[01]	[01]
