# Supplementary material for: Early ontogeny and sequence heterochronies in Leiuperinae frogs (Anura: Leptodactylidae)
Source: PLoS One. 2019 Jun 27;14(6):e0218733. doi: 10.1371/journal.pone.0218733 (PMC6597095; doi:10.1371/journal.pone.0218733)
Supplement: S5 Appendix — (PDF) [file pone.0218733.s005.pdf]

S5 Appendix. Matrix of development sequence for the R-package Pgi2. After the nine position codes are letters, and for non-existent events a Z is used. Species: Oa *O. americanus*, Lb *L. bufonius*, Lc *L. chaquensis*, Lf *L. fuscus*, Lg *L. gracilis*, Ll *L. latinasus*, Lp *L. plaumanni*, Lr *L. latrans*, Pha *Ph.* aff. *albonotatus*, Phb *Ph. albifrons*, Phc *Ph. cicada*, Phf *Ph. fernandezae*, Phg *Ph. gracilis*, Phh *Ph. henselii*, Phi *Ph. biligonigerus*, Pho *Ph. albonotatus*, Phr *Ph. riograndensis*, Phs *Ph. santafecinus*, Phu *Ph. cuvieri*, Phz *Ph. carrizorum*, Plb *Pl. borellii*, Plc *Pl. cordobae*, Pld *Pl. diplolister*, Pli *Pl. bibroni*, Plg *Pl. guayapae*, Pln *Pl. nebulosum*, Plt *Pl. thaul*, Plu *Pl. bufoninum*, Psf *Ps. falcipes*, Psm *Ps. mystacalis* (specimens with oral lower papillation). Events (N = 27): Tail and hind limbs: TB tailbud, TL=BL tail length / body length = 1, HLB hind limb buds, HL26 hind limbs at GS26; Adhesive glands: AG adhesive glands first visible, AGS adhesive glands separated, AGA adhesive glands absent; Gills: 1G first gill pair bud, 1GB first gill pair branched, 2G second gill pair bud, 2GB second gill pair branched, 3G third gill pair bud, GFD gills at full development, OB operculum at gill base, OM operculum medially fused, RGC right gill covered by operculum, LGC left gill covered by operculum, ES spiracle developed; Oral disc and digestive tract: A1 labial tooth ridge A1, A2 labial tooth ridge A2, P1 labial tooth ridge P1, P2 labial tooth ridge P2, P3 labial tooth ridge P3, FP first marginal papillae, MP marginal papillae complete, LOD oral disc fully formed, IC first coil in the digestive tract.

	Oa	*Lb*	*Lc*	*Lf*	*Lg*	*Ll*	*Lp*	*Lr*	*Pha*	*Phb*	*Phc*	*Phf*	*Phg*	*Phh*	*Phi*	*Pho*	*Phr*	*Phs*	*Phu*	*Phz*	*Plb*	*Plc*	*Pld*	*Pli*	*Plg*	*Pln*	*Plt*	*Plu*	*Psf*	*Psm*
TB	1	1	1	1	1	1	1	1	1	1	1	1	1	1	1	1	1	1	1	1	1	1	1	1	1	1	1	1	1	1
TL=BL	2	5	4	7	8	4	6	4	7	6	8	4	1	2	4	4	6	5	4	6	6	5	8	5	8	1	6	9	6	5
HLB	E	B	D	A	C	8	A	D	I	H	H	J	B	C	L	J	I	I	K	F	L	F	E	I	B	6	J	I	H	E
HL26	H	E	F	B	F	C	E	H	J	I	I	K	C	F	M	L	K	L	O	H	L	I	I	K	C	8	M	N	K	G
AG	1	Z	1	Z	Z	Z	Z	1	2	1	1	1	1	1	1	1	1	1	1	1	1	1	1	2	1	1	1	1	1	1
AGS	2	Z	Z	Z	Z	Z	Z	Z	3	2	2	2	1	1	2	2	1	2	2	2	2	2	2	3	2	3	2	2	2	2
AGA	F	Z	H	Z	Z	Z	Z	G	K	E	I	H	C	G	J	K	L	J	M	G	L	K	J	L	J	A	L	P	J	F
1G	2	2	2	2	2	2	2	2	4	2	2	3	1	1	3	3	2	3	3	2	2	3	3	4	3	1	3	3	3	3
1GB	4	4	3	3	3	4	3	3	6	4	4	5	2	2	5	5	4	5	5	4	4	6	5	8	5	2	5	4	5	6
2G	3	3	6	4	4	3	4	5	5	3	3	4	1	1	4	4	3	4	4	3	3	4	4	4	4	1	4	5	4	4
2GB	5	6	8	6	6	4	7	8	7	5	5	8	2	2	6	7	5	6	6	5	5	7	6	8	6	2	8	6	7	7
3G	6	7	9	5	7	5	5	7	8	6	7	Z	2	Z	9	8	8	9	8	6	6	8	7	9	7	2	7	7	Z	Z
GFD	9	9	C	8	9	7	A	9	A	8	C	9	4	4	B	B	C	E	C	9	C	C	B	A	A	4	A	B	8	6
OB	7	7	4	7	5	4	6	4	8	6	6	6	2	3	7	6	7	8	7	4	7	6	7	7	7	2	6	8	5	6
OM	9	A	B	A	A	7	9	B	C	A	A	B	5	7	C	D	B	D	F	9	D	A	B	B	D	4	C	C	A	8
RGC	A	D	E	C	D	A	B	C	F	C	E	D	8	8	F	F	E	F	I	C	F	D	D	C	F	6	D	G	C	B
LGC	C	F	F	D	E	B	D	E	G	D	F	E	9	A	H	G	G	G	J	D	G	G	F	F	G	7	F	L	E	C
ES	D	G	G	E	G	D	F	F	H	F	G	F	A	B	I	I	H	H	L	E	J	I	G	G	H	8	H	M	F	D
A1	7	5	5	6	5	4	6	4	8	7	8	6	2	3	8	9	6	7	9	6	7	7	8	6	8	2	7	A	6	6
A2	9	8	6	A	8	6	7	6	A	8	9	9	4	4	E	B	9	B	B	8	A	B	9	A	E	5	9	D	B	C
P1	8	8	6	A	9	6	7	5	8	7	8	6	2	3	8	9	6	7	A	6	8	B	9	D	E	4	A	H	6	6
P2	7	5	5	6	5	4	6	4	9	8	9	7	3	4	A	A	A	A	9	7	7	7	8	6	8	2	7	A	9	7
P3	F	8	7	B	9	6	8	6	B	9	D	G	6	5	Z	C	Z	Z	E	B	E	E	B	E	Z	Z	E	J	E	D
FP	8	A	A	9	9	7	8	5	A	8	A	A	4	6	A	B	C	B	D	7	9	9	A	8	9	3	B	E	9	A
MP	B	C	B	B	C	A	D	A	E	G	G	F	7	D	G	H	F	I	H	D	H	H	C	H	G	6	I	K	G	D
LOD	G	H	G	F	H	E	G	H	J	G	J	I	C	E	K	L	J	K	N	E	I	J	H	J	I	9	K	O	I	E
IC	9	9	9	9	B	9	9	8	D	B	B	C	6	9	D	E	D	C	G	A	B	C	B	D	B	4	G	F	D	9
