# Supplementary material for: Early ontogeny and sequence heterochronies in Leiuperinae frogs (Anura: Leptodactylidae)
Source: PLoS One. 2019 Jun 27;14(6):e0218733. doi: 10.1371/journal.pone.0218733 (PMC6597095; doi:10.1371/journal.pone.0218733)

S1 Figure. Embryonic measurements and morphological features recorded. (A) Measurements taken in tailbud stage embryos. (B-C) Measurements taken in gills and adhesive glands. (D) Ventral view of a Stage 23 embryo, showing transient embryonic structures and the oral disc. (E) Detail of an adhesive gland at full development. (F) Detail of a developing oral disc showing the jaw sheaths, the anterior and posterior lower tooth ridges, and marginal papillae with a dorsal gap. (G) Detail of epidermal ciliated cells. (H) Detail of an individual labial teeth showing cusp pattern. (I) Detail of the cephalic region of a tailbud stage embryo, showing the area of the hatching gland.

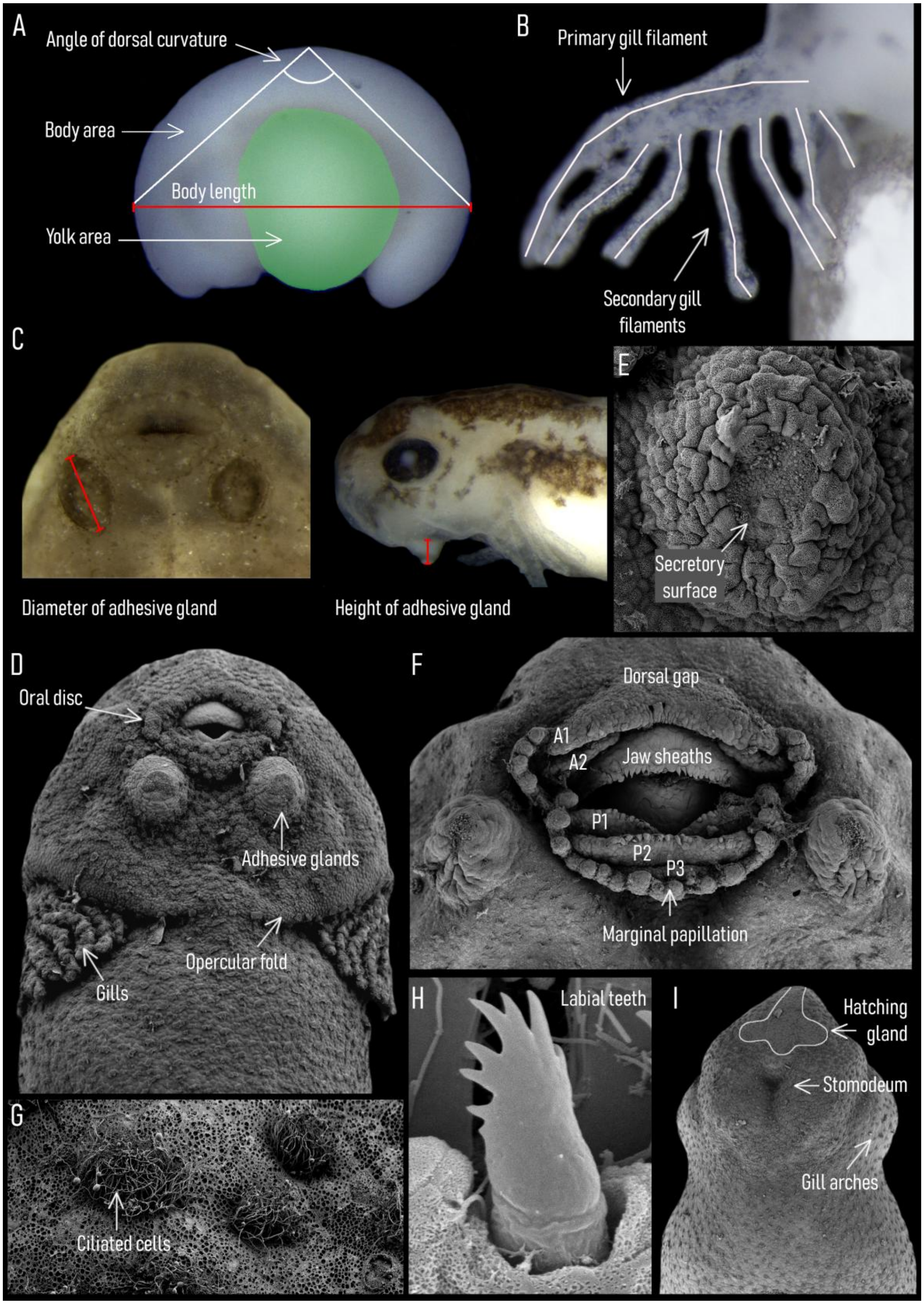

Supplement: S1 Fig — (PDF) [file pone.0218733.s006.pdf]
