# Supplementary material for: Early ontogeny and sequence heterochronies in Leiuperinae frogs (Anura: Leptodactylidae)
Source: PLoS One. 2019 Jun 27;14(6):e0218733. doi: 10.1371/journal.pone.0218733 (PMC6597095; doi:10.1371/journal.pone.0218733)
Supplement: S1 Table — (PDF) [file pone.0218733.s007.pdf]

S1 Table. Summary of morphological characters in leiuperine embryos. Features and measurements at tailbud stage, external gills, and adhesive glands are shown. Data were registered from personal observations, or from literature when available. In addition to the 8 species used as outgroups in all analyses, we include here data for leiuperine *Engystomops* and bufonid *Melanophryniscus*, to compare with other kyphotic embryos with information available. Abbreviations: Tailbud stage: BL body length, YS yolk shape, YA yolk area, P pigmentation, YA/BA proportion of yolk area regarding body area, C semicircular, O oblong; Dorsal curvature: A angle, CeK kyphosis in cephalic region, MeK kyphosis in medial region, CaK kyphosis in caudal region; Gills: Gill pairs (1p–3p), FN gill filaments number per side, FP length of the primary gill filament, FS length of the secondary gill filament, FA length average of all filaments of the third gill pair; Adhesive glands: D maximum diameter at full development, H height at full development, EL embryo total length; Tadpole (data from the original description): TL total length; BP body proportion. Data are shown in millimeters, except for the yolk area (mm^2), yolk proportion (%), angle of dorsal curvature (degrees), and body proportion (% of TL). Superindexes in species: [*]species with intraspecific variation in the number of external gills pairs; [#]species with intraspecific variation of the length of gills at full development (largest values are shown; data taken from literature [a]Romero-Carvajal et al., 2009, [b]Bernabé Andrade Cahueñas , 2015, [c]Nokhbatolfoghahai & Downie, 2008, [d]Leon-Ochoa & Donoso-Barros, 1970, [e]Grosso et al., 2017, [f]Vera Candioti et al., 2016, [1]Cannatella & Duellman, 1984; [2]Ron et al., 2005; [3]Kenny, 1969; [4]Borteiro & Kolenc, 2007; [5]Perotti &Céspedez, 1999; [6]Langone, 1989; [7]Oliveira et al., 2010; [8]Kehr et al., 2004 (as *Ph. albonotatus*); [9]Perotti 1997 (as *Ph. cuqui*); [10]Vieira & Arzabe, 2008; [11]Alcalde et al.,2006; [12]Kolenc et al., 2006; [13]Kolenc et al., 2009; [14]Otero et al., 2013; [15]Barrasso et al., 2013; [16]Galvani et al., 2012; [17]Rada, 1981;[18]Peixoto 1982; [19]Laufer & Berreneche, 2008; [20]Guiaretta & Facure, 2009.

	Tailbud stage					Dorsal curvature				1p			2p			3p		Adhesive glands			Tadpole	
Species	BL	YS	YA	P	YA/BA	A	CeK	MeK	CaK	FN	FP	FS	FN	FP	FS	FN	FA	D	H	EL	TL	BP
Engystomops coloradorum [a]	1.70	C	0.73	no	42.4	64	yes	yes	yes	6	?	?	5	?	?	-	-	?	?	?	19.3	37.3[1]
En. guayaco [b]	1.5	C	0.42	no	32.8	57	yes	yes	yes	?	?	?	?	?	?	?	?	?	?	?	18.9	37.6[2]
En. pustulosus [c*]	?	?	?	no	?	?	?	?	?	6	?	?	5	?	?	-	-	?	?	?	30	33.3[3]
En. randi [a]	?	?	?	no	?	?	?	?	?	6	?	?	5	?	?	-	-	?	?	?	?	?
Physalaemus biligonigerus	1.65	C	0.52	no	40.0	75	yes	yes	yes	6	0.68	0.30	5	0.35	0.21	1	0.04	0.10	0.06	3.80	23.6	43.2[4]
Ph. riograndensis [#]	1.36	?	0.46	yes	48.0	64	?	?	?	6-7	1.04	0.44	4	0.45	0.25	5	0.07	0.14	0.12	4.79	14.7	39.46[4]
Ph. santafecinus [#]	1.43	C	0.57	no	47.0	71	yes	yes	yes	10-12	0.69	0.41	10-12	0.60	0.35	10-12	0.08	0.12	0.13	4.10	22.0	34[5]

	Tailbud stage					Dorsal curvature				1p			2p			3p		Adhesive glands			Tadpole	
Species	BL	YS	YA	P	YA/BA	A	CeK	MeK	CaK	FN	FP	FS	FN	FP	FS	FN	FA	D	H	EL	TL	BP
Ph. carrizorum	1.90	C	0.91	no	46.8	80	yes	no	yes	9-10	0.97	0.37	9	0.68	0.36	7	0.7	0.14	0.10	5.12	?	?
Ph. gracilis	?	?	?	no	?	?	?	?	?	6-7	0.75	0.41	6-7	0.58	0.39	6-7	0.14	0.18	0.16	6.18	30.7	50[6]
Ph. albifrons	1.40	C	0.38	no	33.7	46	yes	yes	yes	8-9	0.79	0.31	6-7	0.52	0.23	6-7	0.07	0.12	0.06	3.61	19.4	35.6[7]
Ph. aff. *albonotatus*	1.53	C	0.48	no	42.5	66	yes	yes	yes	8-9	0.73	0.30	6-7	0.54	0.33	2-4	0.10	0.13	0.12	4.30	24.2	41.3[8]
Ph. albonotatus	1.50	O	0.44	no	38.0	62	yes	yes	yes	8	0.91	0.36	6-7	0.86	0.31	5	0.06	0.08	0.09	3.34	21.9	35.6[9]
Ph. cuvieri	1.55	C	0.77	no	41.5	63	yes	yes	yes	7-8	0.72	0.21	6-7	0.46	0.22	2-3	0.20	0.09	0.14	3.98	26[6]	?
Ph. cicada	?	C	?	yes	?	?	?	?	?	8-9	0.84	0.36	7-8	0.66	0.32	4-5	0.12	0.09	0.07	3.41	17.5	42.6[10]
Ph. fernandezae	?	?	?	no	?	?	?	?	?	3-4	0.30	0.08	1-2	0.08	-	-	-	0.14	0.10	3.78	26.8	32.5[11]
Ph. henselii	?	?	?	no	?	?	?	?	?	2-3	0.25	0.10	1-2	0.09	-	-	-	0.05	0.13	3.73	25.2	35.3[12]
Pleurodema bibroni	?	?	?	yes	?	?	?	?	?	2-3	0.18	0.05	2-3	0.12	0.05	2-3	0.05	0.24	0.12	5.97	35.4	41.8[13]
Pl. cordobae	2.70	O	1.08	yes	40.5	160	no	no	no	5-6	0.39	0.19	5-6	0.25	0.11	5-6	0.09	0.31	0.15	6.64	38.98	41,7[14]
Pl. bufoninum	2.30	O	1.26	yes	46.7	104	no	no	yes	6-7	0.53	0.18	5-6	0.28	0.11	3	0.06	0.26	0.10	5,78	42.04	43.8[15]
Pl. thaul	2.41	O	1.23	yes	45.8	96	yes	no	yes	4	0.35	0.14	3	0.24	0.09	3-4	0.07	0.26	0,15	6.02	44.93	36.5[15]
Pl. guayapae	1.65	O	0.76	yes	38.7	120	?	?	?	9-10	0.68	0.26	9-10	0.51	0.10	6	0.07	0.18	0.09	3.86	?	?
Pl. nebulosum	?	?	?	yes	?	?	yes	no	yes	8-9	0.74	0.39	12-13	0.40	0.20	7-8	0.15	?	?	?	20.7	43[16]
Pl. brachyops [d]	1.65	O	?	no	?	?	no	no	no	?	?	?	?	?	?	?	?	?	?	?	26.2[17]	?
Pl. borellii	2	O	0.95	yes	41.0	95	no	no	yes	7-8	0.55	0.23	9-10	0.30	0.14	7-8	0.07	0.23	0.12	4.03	25.5	?
Pl. diplolister	1.70	O	0.48	yes	41.0	139	no	no	no	6-7	0.80	0.40	6-7	0.62	0.25	6-7	0.06	0.19	0.05	3.04	25	36[18]
Pseudopaludicola falcipes	1.06	O	0.32	yes	30.2	143	no	no	no	2-3	0.24	0.06	2-3	0.14	0.05	-	-	0.08	0.05	4.50	21.11	36.5[19]
Ps. mystacalis	2.10	?	?	yes	?	?	?	?	?	2	0.24	0.13	1	0,16	-	-	-	0.09	0.06	3.71	22.5	38.5[20]

	Tailbud stage					Dorsal curvature				1p			2p			3p		Adhesive glands			Tadpole	
Species	BL	YS	YA	P	YA/BA	A	CeK	MeK	CaK	FN	FP	FS	FN	FP	FS	FN	FA	D	H	EL	TL	BP
Leptodactylus bufonius [e]	2.50	C	2.34	no	59.0	112	yes	no	yes	4-5	0.60	0.3-0.6	5-6	0.10	?	5	0.10	-	-	-		
Le. fuscus [e]	2.00	C	1.93	no	64.0	129	yes	no	yes	4-4	0.84	0.2-0.6	5-6	0.10	?	5	0.10	-	-	-		
Le. gracilis [e]	2.90	C	3.40	no	63.0	116	yes	no	yes	4-5	0.66	0.45	5-6	0.10	?	5	0.10	-	-	-		
Le. latinasus [e]	1.90	C	1.25	no	50.0	102	yes	no	yes	5	0.60	0.3-0.6	5-6	0.10	?	5	0.10	-	-	-		
Le. plaumanni [e]	2.50	C	?	no	57.0	96	yes	no	yes	4-5	0.66	0.3-0.6	5-6	0.10	?	5	0.10	-	-	-		
Le. chaquensis [e]	2.15	O	?	yes	39.0	145	yes	no	yes	17-20	1.50	0.3-0.7	6	?	?	4	?	?	?	?		
Le. latrans [e]	2.00	C	1.03	yes	44.0	100	yes	no	yes	14-20	1.50	0.3-0.7	4	?	?	4	?	?	?	?		
Odontophrynus americanus	1.36	C	0.38	yes	30.4	83	yes	no	yes	?	?	?	?	?	?	?	?	?	?	?		
Melanophryniscus atroluteus [f]	2.38	C	0.91	yes	36.0	107	yes	no	yes	?	?	?	?	?	?	?	?	?	?	?		
Me. krauckzuki [f]	1.94	C	0.65	yes	25.0	63	yes	yes	yes	?	?	?	?	?	?	?	?	?	?	?		
Me. sanmartini [f]	1.76	C	0.99	yes	48.0	58	yes	no	yes	?	?	?	?	?	?	?	?	?	?	?		
