# Supplementary material for: Early ontogeny and sequence heterochronies in Leiuperinae frogs (Anura: Leptodactylidae)
Source: PLoS One. 2019 Jun 27;14(6):e0218733. doi: 10.1371/journal.pone.0218733 (PMC6597095; doi:10.1371/journal.pone.0218733)
Supplement: S2 Table — (PDF) [file pone.0218733.s008.pdf]

S2 Table. Sequences of developmental events in species of Leiuperinae. *Physalaemus*: A–C) *P. biligonigerus* group, D–E) *P. cuvieri* group, F–G) *P. henselii* group, H) *P. gracilis* group; *Pleurodema*: I–J) *P. bibroni* group, K) *P. borelli* group, L–M) *P. nebulosum* clade, N–O) *P. thaul* clade; *Pseudopaludicola*: P–Q) *P. falcipes* group. Events (N = 34) are grouped in rows per structure, and hatching and the emergence of first labial teeth are indicated in additional rows, to highlight their intraspecifically variable occurrence. Columns across structures indicate simultaneous events. Since the X-axis does not represent time, shorter trajectories are to be interpreted as a consequence of poor sampling and high event synchrony. Events: H hatching (orange); Tail and limbs (reds): TB tailbud, FB caudal fin buds, TL=BL tail length / body length = 1, TL=2BL tail length / body length = 2, HLB hind limb buds, HL26 hind limbs at GS26; Adhesive glands (violets): AG adhesive glands first visible, AGS adhesive glands separated, AGA adhesive glands absent; Gills (greens): 1G first gill pair bud, 1GB first gill pair branched, 2G second gill pair bud, 2GB second gill pair branched, 3G third gill pair bud, GFD gills at full development, OB operculum at gill base, OM operculum medially fused, RGC right gill covered by operculum, LGC left gill covered by operculum, ES spiracle developed; Oral disc and digestive tract (yellows): A1 labial tooth ridge A1, A2 labial tooth ridge A2, P1 labial tooth ridge P1, P2 labial tooth ridge P2, P3 labial tooth ridge P3, CP marginal papillae at commissures, MEP marginal papillae medial in mental region, LP marginal papillae lateral in mental region, MP marginal papillae with larval configuration; LOD oral disc fully formed, IC first coil in digestive tract; AF onset of active feeding; TE first labial teeth (blue).

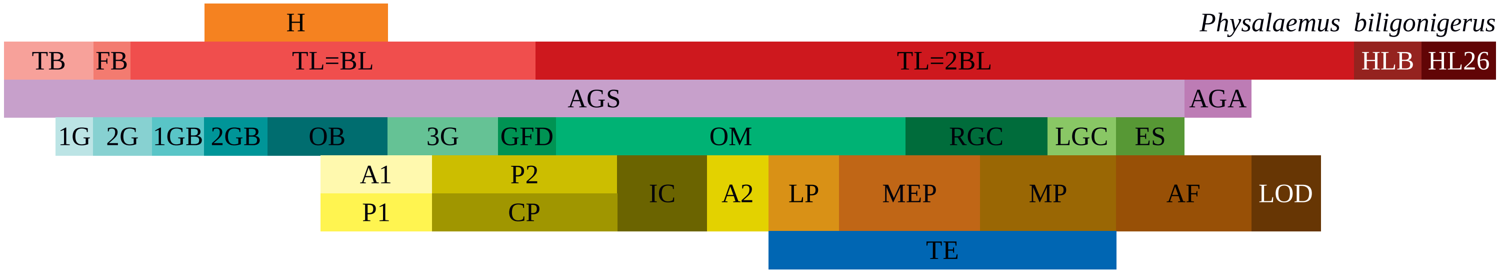

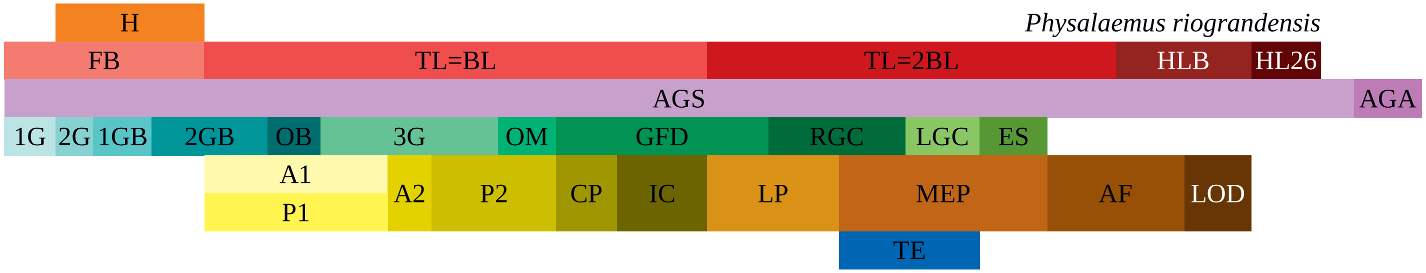

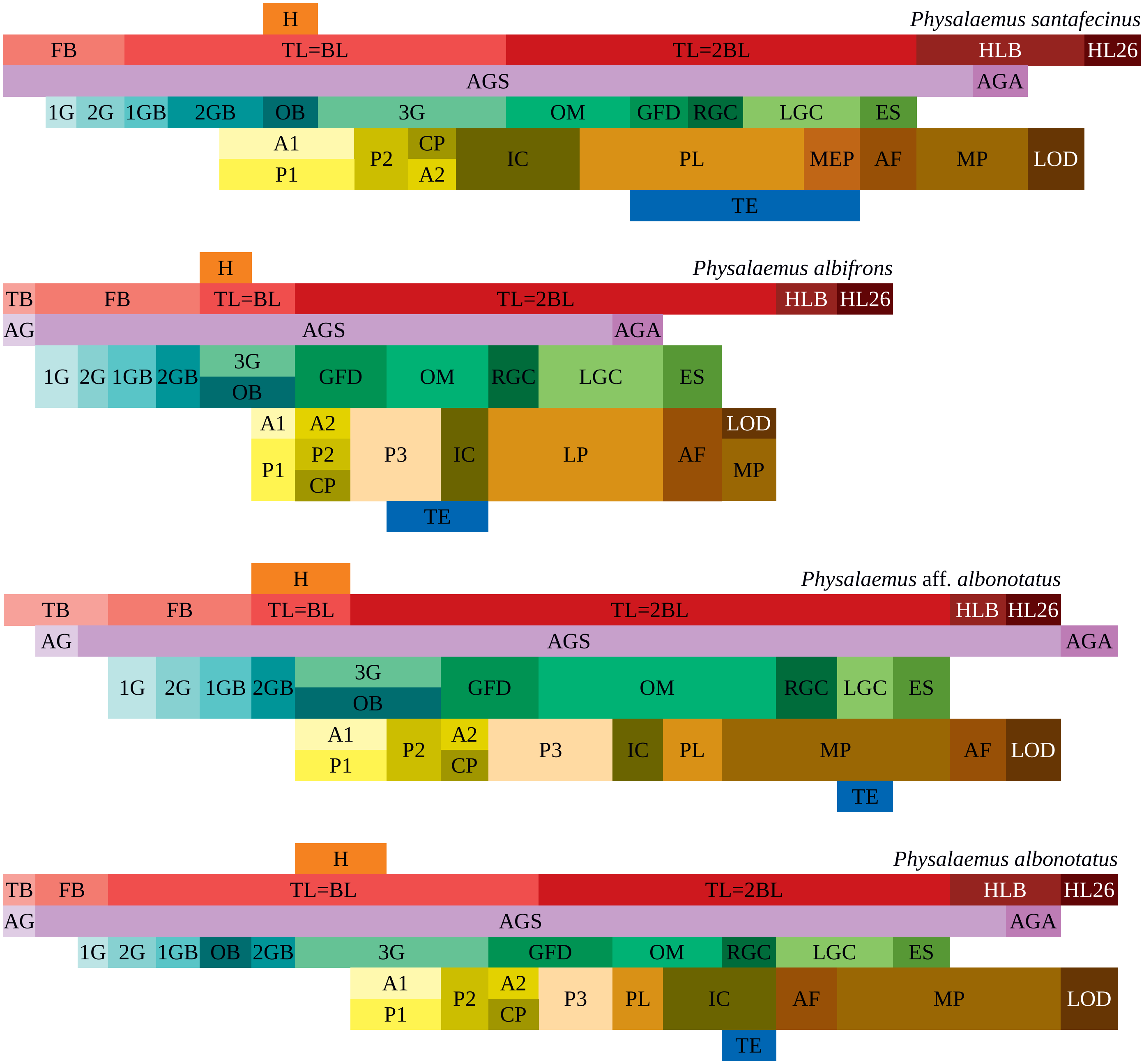

Physalaemus santafecinus
H
FB TL=BL TL=2BL HLB HL26
AGS AGA
1G 2G 1GB 2GB OB 3G OM GFD RGC LGC ES
A1 P1 P2 CP A2 IC PL MEP AF MP LOD
TE
Physalaemus albifrons
H
TB FB TL=BL TL=2BL HLB HL26
AG AGS AGA
1G 2G 1GB 2GB 3G OB GFD OM RGC LGC ES
A1 P1 A2 P2 CP P3 IC LP AF LOD MP
TE
Physalaemus aff. albonotatus
H
TB FB TL=BL TL=2BL HLB HL26
AG AGS AGA
1G 2G 1GB 2GB 3G OB GFD OM RGC LGC ES
A1 P1 P2 A2 CP P3 IC PL MP AF LOD
TE
Physalaemus albonotatus
H
TB FB TL=BL TL=2BL HLB HL26
AG AGS AGA
1G 2G 1GB OB 2GB 3G GFD OM RGC LGC ES
A1 P1 P2 A2 CP P3 PL IC AF MP LOD
TE

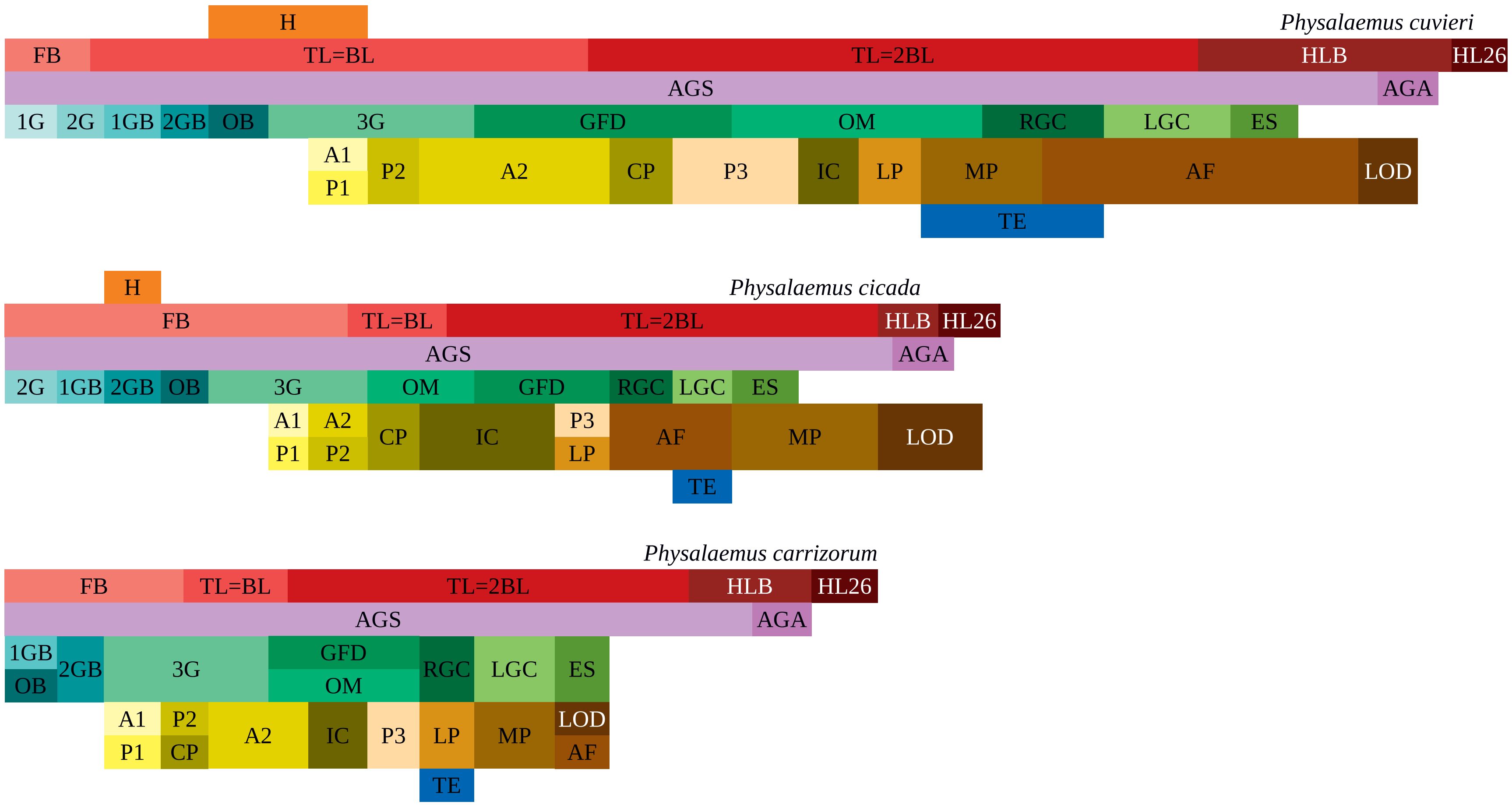
Physalaemus cuvieri
H
FB
TL=BL
TL=2BL
HLB
HL26
AGS
AGA
1G
2G
1GB
2GB
OB
3G
GFD
OM
RGC
LGC
ES
A1
P1
P2
A2
CP
P3
IC
LP
MP
AF
LOD
TE
Physalaemus cicada
H
FB
TL=BL
TL=2BL
HLB
HL26
AGS
AGA
2G
1GB
2GB
OB
3G
OM
GFD
RGC
LGC
ES
A1
A2
P1
P2
CP
IC
P3
LP
AF
MP
LOD
TE
Physalaemus carrizorum
FB
TL=BL
TL=2BL
HLB
HL26
AGS
AGA
1GB
OB
2GB
3G
GFD
OM
RGC
LGC
ES
A1
P2
P1
CP
A2
IC
P3
LP
MP
LOD
AF
TE

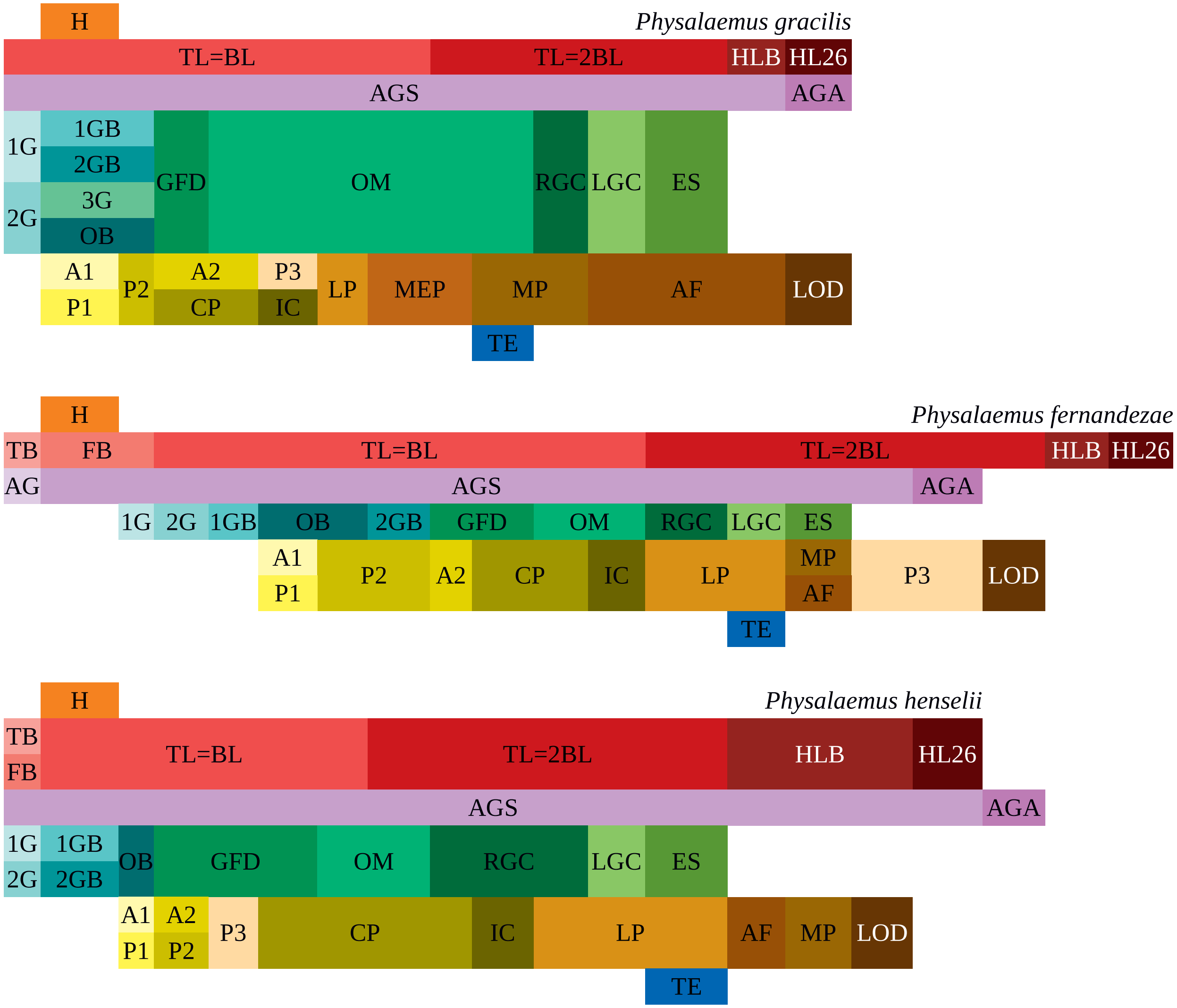
Physalaemus gracilis
H
TL=BL
TL=2BL
HLB
HL26
AGS
AGA
1G
1GB
2GB
2G
3G
OB
GFD
OM
RGC
LGC
ES
A1
P1
P2
A2
CP
P3
IC
LP
MEP
MP
AF
LOD
TE
Physalaemus fernandezae
H
TB
FB
TL=BL
TL=2BL
HLB
HL26
AG
AGS
AGA
1G
2G
1GB
OB
2GB
GFD
OM
RGC
LGC
ES
A1
P1
P2
A2
CP
IC
LP
MP
AF
P3
LOD
TE
Physalaemus henselii
H
TB
FB
TL=BL
TL=2BL
HLB
HL26
AGS
AGA
1G
2G
1GB
2GB
OB
GFD
OM
RGC
LGC
ES
A1
P1
A2
P2
P3
CP
IC
LP
AF
MP
LOD
TE

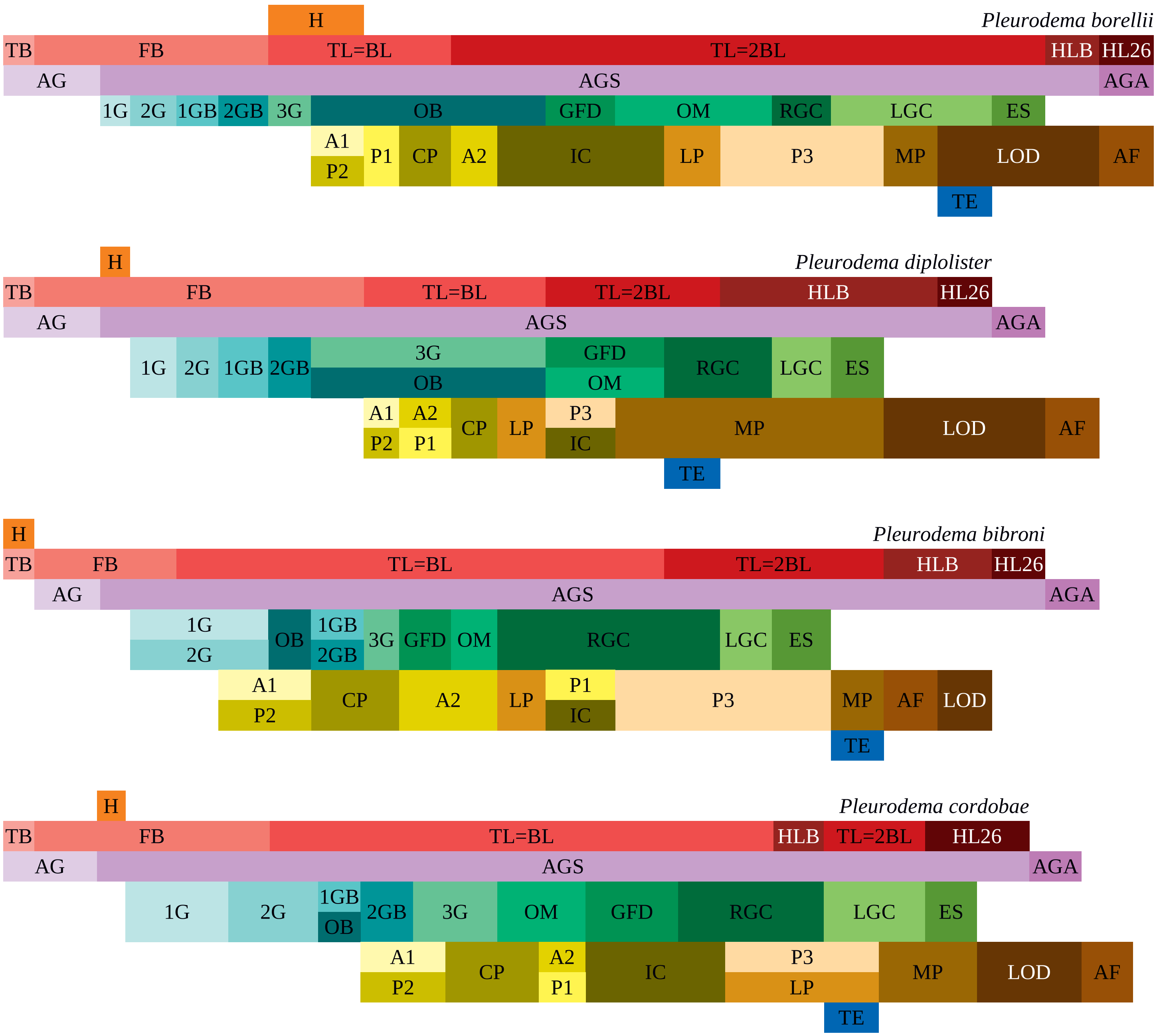

Pleurodema borellii
H
TB FB TL=BL TL=2BL HLB HL26
AG AGS AGA
1G 2G 1GB 2GB 3G OB GFD OM RGC LGC ES
A1 P2 P1 CP A2 IC LP P3 MP LOD AF
TE
Pleurodema diplolister
H
TB FB TL=BL TL=2BL HLB HL26
AG AGS AGA
1G 2G 1GB 2GB 3G OB GFD OM RGC LGC ES
A1 P2 A2 P1 CP LP P3 IC MP LOD AF
TE
Pleurodema bibroni
H
TB FB TL=BL TL=2BL HLB HL26
AG AGS AGA
1G 2G OB 1GB 2GB 3G GFD OM RGC LGC ES
A1 P2 CP A2 LP P1 IC P3 MP AF LOD
TE
Pleurodema cordobae
H
TB FB TL=BL HLB TL=2BL HL26
AG AGS AGA
1G 2G 1GB OB 2GB 3G OM GFD RGC LGC ES
A1 P2 CP A2 P1 IC P3 LP MP LOD AF
TE

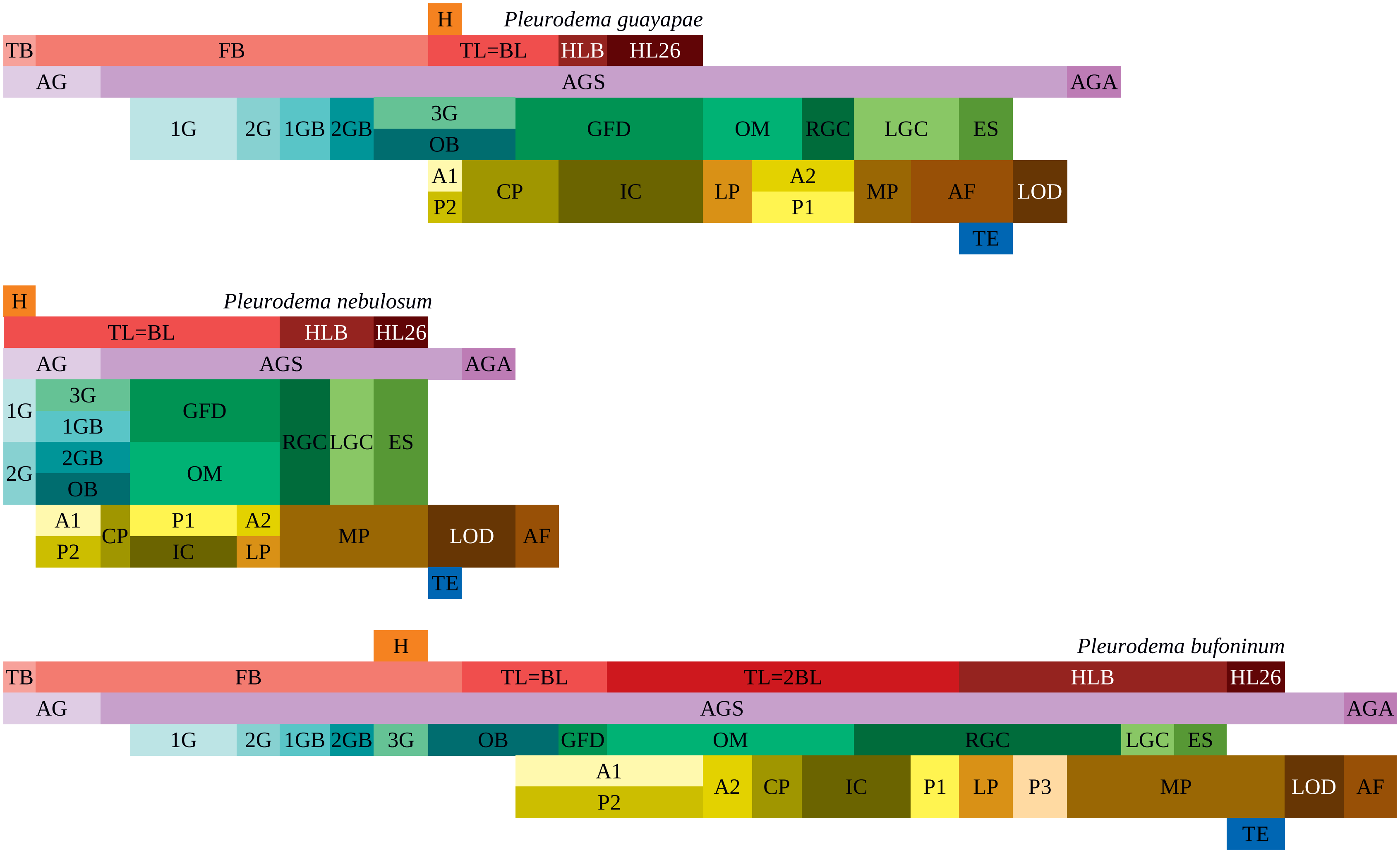
Pleurodema guayapae
H
TB FB TL=BL HLB HL26
AG AGS AGA
1G 2G 1GB 2GB 3G OB GFD OM RGC LGC ES
A1 P2 CP IC LP A2 P1 MP AF LOD
TE
Pleurodema nebulosum
H
TL=BL HLB HL26
AG AGS AGA
1G 3G 1GB GFD RGC LGC ES
2G 2GB OB OM
A1 P2 CP P1 IC A2 LP MP LOD AF
TE
Pleurodema bufoninum
H
TB FB TL=BL TL=2BL HLB HL26
AG AGS AGA
1G 2G 1GB 2GB 3G OB GFD OM RGC LGC ES
A1 P2 A2 CP IC P1 LP P3 MP LOD AF
TE

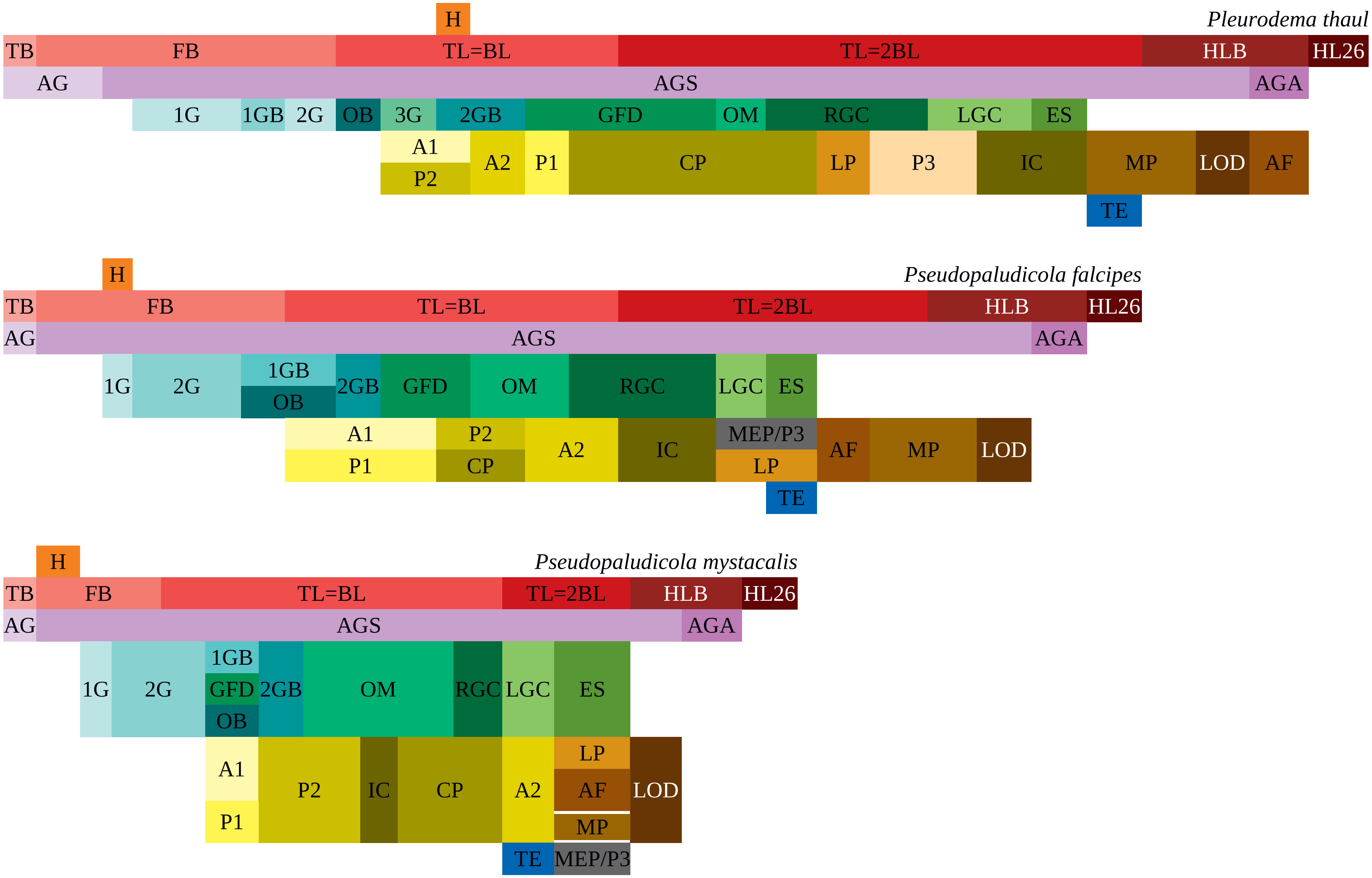
Pleurodema thaul
H
TB FB TL=BL TL=2BL HLB HL26
AG AGS AGA
1G 1GB 2G OB 3G 2GB GFD OM RGC LGC ES
A1 P2 A2 P1 CP LP P3 IC MP LOD AF
TE
Pseudopaludicola falcipes
H
TB FB TL=BL TL=2BL HLB HL26
AG AGS AGA
1G 2G 1GB OB 2GB GFD OM RGC LGC ES
A1 P1 P2 CP A2 IC MEP/P3 LP AF MP LOD
TE
Pseudopaludicola mystacalis
H
TB FB TL=BL TL=2BL HLB HL26
AG AGS AGA
1G 2G 1GB GFD OB 2GB OM RGC LGC ES
A1 P1 P2 IC CP A2 LP AF MP LOD
TE MEP/P3
